# ACVR2A attenuation impacts lactate production and hyperglycolytic conditions attracting regulatory T cells in hepatocellular carcinoma

**DOI:** 10.1016/j.xcrm.2025.102038

**Published:** 2025-03-25

**Authors:** Koya Yasukawa, Shu Shimada, Yoshimitsu Akiyama, Tomohiko Taniai, Yosuke Igarashi, Shu Tsukihara, Yoshiaki Tanji, Kentaro Umemura, Atsushi Kamachi, Atsushi Nara, Masahiro Yamane, Keiichi Akahoshi, Akira Shimizu, Yuji Soejima, Minoru Tanabe, Shinji Tanaka

**Affiliations:** 1 Department of Molecular Oncology, Graduate School of Medicine, Tokyo Medical and Dental University, Tokyo 113-8519, Japan; 2 Division of Gastroenterological, Hepato-Biliary-Pancreatic, Transplantation and Pediatric Surgery, Department of Surgery, Shinshu University School of Medicine, Matsumoto 390-8621, Japan; 3 Division of Hepatobiliary and Pancreas Surgery, Department of Surgery, The Jikei University School of Medicine, Tokyo 105-8471, Japan; 4 Department of Surgery, The Jikei University School of Medicine, Tokyo 105-8471, Japan; 5 Department of Hepato-Biliary-Pancreatic Surgery, Tokyo Medical and Dental University, Graduate School of Medicine, Tokyo Medical and Dental University, Tokyo 113-8519, Japan

**Keywords:** ACVR2A, hepatocellular carcinoma, PD-1, immunotherapy, lactate, MCT4, regulatory T cell, LDHA

## Abstract

Although ACVR2A mutations are prevalent in non-viral hepatocellular carcinomas (HCCs), the underlying mechanism remains unelucidated. Our molecular investigation reveals that ACVR2A impairment induces hyperglycolysis through the inactivation of the SMAD signaling pathway. Using syngeneic transplantation models and human clinical samples, we clarify that ACVR2A-deficient HCC cells produce and secrete lactate via the upregulation of lactate dehydrogenase A (LDHA) and monocarboxylate transporter 4 (MCT4) expression levels, which promotes regulatory T (Treg) cell accumulation and then acquires resistance to immune checkpoint inhibitors. Remarkably, genetic knockdown and pharmacological inhibition of MCT4 ameliorate the high-lactate milieu in ACVR2A-deficient HCC, resulting in the suppression of intratumoral Treg cell recruitment and the restoration of the sensitivity to PD-1 blockade. These findings furnish compelling evidence that lactate attenuates anti-tumor immunity and that therapeutics targeting this pathway present a promising strategy for mitigating immunotherapy resistance in ACVR2A-deficient HCC.

## Highlights

• ACVR2A inactivation increases LDHA and MCT4 expression, promoting lactate secretion
• Lactate recruits Foxp-3+ Treg cells, which evade CD8^+^ T cells and resist PD-1 blockade
• MCT4 inhibition reversed the sensitivity to immunotherapy

## Graphical abstract

**Figure:**
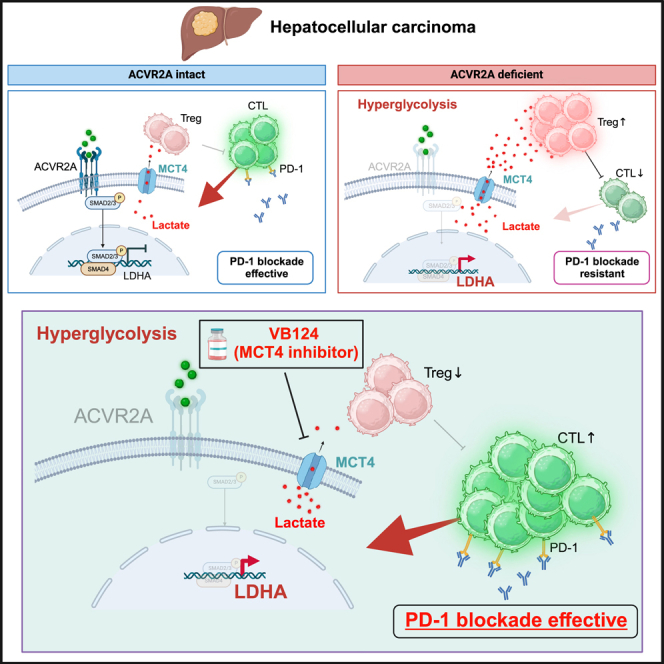

## Teaser

Yasukawa et al. demonstrate that ACVR2A inactivation in HCC induces hyperglycolysis by disrupting SMAD4 signaling, leading to LDHA upregulation, lactate secretion, and regulatory T cell recruitment. This metabolic reprogramming promotes immune evasion and PD-1 blockade resistance. Targeting MCT4 restores immune sensitivity, suggesting a potential therapeutic approach for ACVR2A-deficient HCC.

## Introduction

Hepatocellular carcinoma (HCC) is the most predominant form of primary liver cancer,^1^ with various risk factors including infection with hepatitis B virus (HBV) and hepatitis C virus (HCV), alcohol abuse, and metabolic syndrome.^2^ Previous studies have identified signaling pathways playing important roles in the initiation and progression of HCC, such as the Wnt/β-catenin,^3^^,^^4^ Notch,^5^^,^^6^ PI3K/AKT/mTOR,^7^^,^^8^ and transforming growth factor β/SMAD signaling pathways.^9^^,^^10^ In recent years, the prevalence of metabolism-associated liver cancer^11^ and the emergence of immunotherapy with immune checkpoint inhibitors have sparked considerable discussion about molecular mechanisms and therapeutic strategies,^12^^,^^13^^,^^14^ necessitating further investigation.

Pinyol et al.^15^ have reported frequent mutations of *ACVR2A*, encoding activin A receptor type 2A, in HCC linked to non-alcoholic steatohepatitis (NASH-HCC), which is known as an HCC subgroup resistant to anti-PD-1 therapy, and have also addressed that *ACVR2A* silencing accelerates cell proliferation in HCC cells. However, the detailed molecular mechanism and the biological function of the activin/SMAD signaling pathway in HCC have not fully been elucidated, since activin works as a negative regulator of hepatocyte growth.^16^ *ACVR2A* mutations are frequently detected in microsatellite instability-high subtypes of colorectal cancer and gastric cancer,^17^ and ACVR2A inactivation is associated with unfavorable prognosis in colorectal cancer^18^ in spite of the inverse relationship in gastric cancer.^19^ Nevertheless, the precise functional implications of ACVR2A in carcinogenesis remain unclear.

In the present study, we established *ACVR2A*-knockout (KO) cells from both human and mouse HCC cell lines, in which glycolysis was strongly enhanced and lactate was increasingly produced, and elucidated the significant relationship between *ACVR2A* depletion and regulatory T (Treg) cell infiltration by histopathological analysis of human HCC samples. Considering two recent papers on the stimulation of glycolysis following loss of SMAD4 in pancreatic cancer^20^ and the accumulation of Treg cells within lactate-rich microenvironment in liver metastatic cancer,^21^ we discovered that ACVR2A inactivation upregulated lactate dehydrogenase A (LDHA) expression via SMAD signal transduction and promoted lactate secretion through monocarboxylate transporter (MCT)4, resulting in immune evasion and extrinsic resistance to immune checkpoint blockade by Treg cell recruitment.

## Results

### Clinical and biological impacts of ACVR2A inactivation on HCC

**Figure 1:**
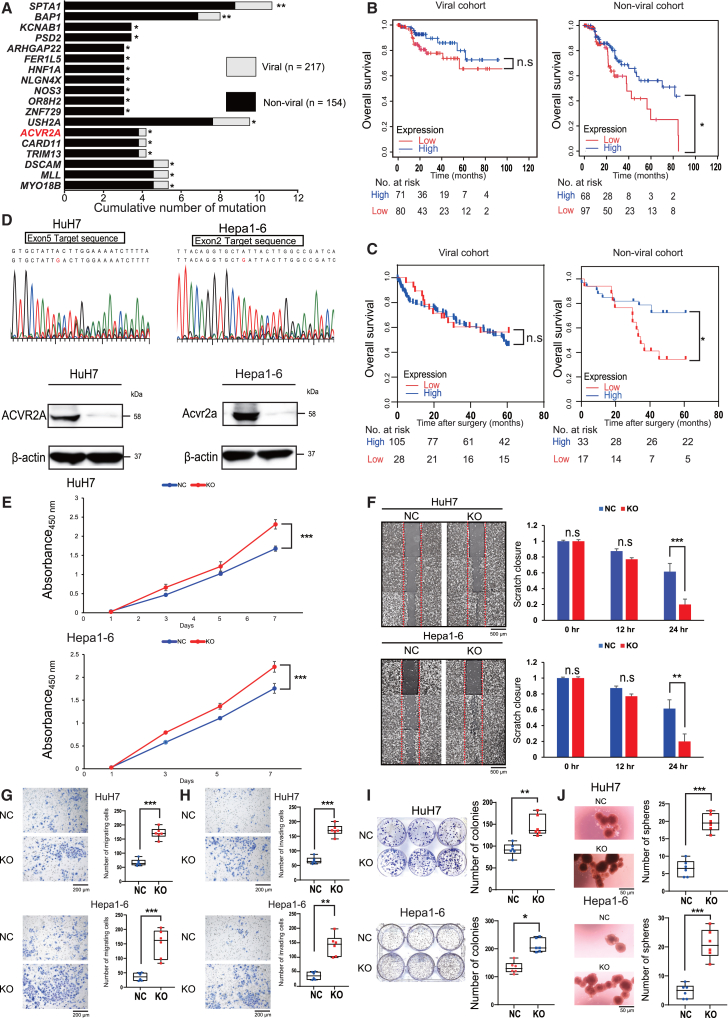
Impacts of ACVR2A attenuation on patient outcomes and biological functions of HCC (A) Cumulative numbers of gene mutations in human viral and non-viral HCC (*N* = 217 and 154, respectively). The 18 genes specifically mutated in non-viral HCC cases are ranked in ascending order of statistical significance. The *p* value was calculated by χ2 test. (B and C) Kaplan-Meier curves of OS in patients with *ACVR2A*-high and -low HCC in the TCGA (B) and Tokyo Medical and Dental University (TMDU) (C) cohorts. The *p* value was calculated by the log rank test. (D) Sequencing analysis (upper panel) and western blot analysis (lower panel) of ACVR2A in HuH7 and Hepa1-6 cells. β-Actin was used as an internal control. (E–J) Proliferation (E), wound healing (F), migration (G), invasion (H), colony formation (I), and sphere formation (J) assays of HuH7 KO and Hepa1-6 KO cells. Representative photo images in each assay were included. The *p* value was calculated by Welch’s *t* test, and data are the mean ± SD (E and F). The *p* value was calculated by Mann-Whitney *U* test, and boxes represent the 25th, 50th, and 75th percentiles (G–J). The scale bar represents 500 (F) or 200 μm (G, H, and J). n.s, not significant; ∗*p* < 0.05, ∗∗*p* < 0.01, ∗∗∗*p* < 0.001.

We utilized genomic and clinical data from 371 HCC patients provided by The Cancer Genome Atlas (TCGA) Research Network to compare the mutation rates of 14,881 genes between viral (HBV- and HCV-related) and non-viral HCC cases and detected 18 genes specifically mutated in non-viral HCC cases (Figure 1A). We subsequently conducted comparative analysis of overall survival (OS) between the high- and low-expression groups in each gene using RNA sequencing (RNA-seq) data from the TCGA cohort (Figure 1B). Notably, *ACVR2A*, *PSD2*, and *CARD11* met the criteria that there was no significant difference in OS between the two groups within the viral cohort, while the low-expression group closely correlated with poor prognosis in the non-viral cohort (Figure S1 and Table S1). Among these genes, we focused on *ACVR2A* as a gene specifically mutated (*p* = 0.027) and associated with unfavorable outcomes (*p* = 0.020) in non-viral HCC cases. The similar findings on patient survival were also observed when examining 183 microarray data of clinical samples surgically resected in our institution (Figure 1C).

Two human HCC cell lines, HuH7 and PLC/PRF/5, and two mouse HCC cell lines, Hepa1-6 and 3H3-Pten-KO, were used for further analysis due to the high mRNA and protein expression levels of ACVR2A (Figure S2A). We performed knockdown (KD) analysis with each two kinds of small interfering RNA (siRNA) for human and mouse *ACVR2A* and confirmed the downregulation of *ACVR2A* at the mRNA and protein levels (Figures S2B–S2D). *ACVR2A* silencing enhanced the cell proliferation and colony formation capacity (Figures S2E and S2F) as previously described.^15^ Next, the CRISPR-Cas9 system was employed to generate *ACVR2A*-KO cells in HuH7 and Hepa1-6 cells, and the frameshift mutation and protein depletion of ACVR2A were validated by Sanger sequencing analysis and western blot analysis, respectively (Figure 1D). Proliferation, wound healing, migration, invasion, colony formation, and sphere formation assays were carried out, all of which demonstrated an augmented malignant phenotype within the KO cells compared to the negative control (NC) cells (Figures 1E–1J).

### ACVR2A inactivation induces glycolysis with lactate production in HCC

**Figure 2:**
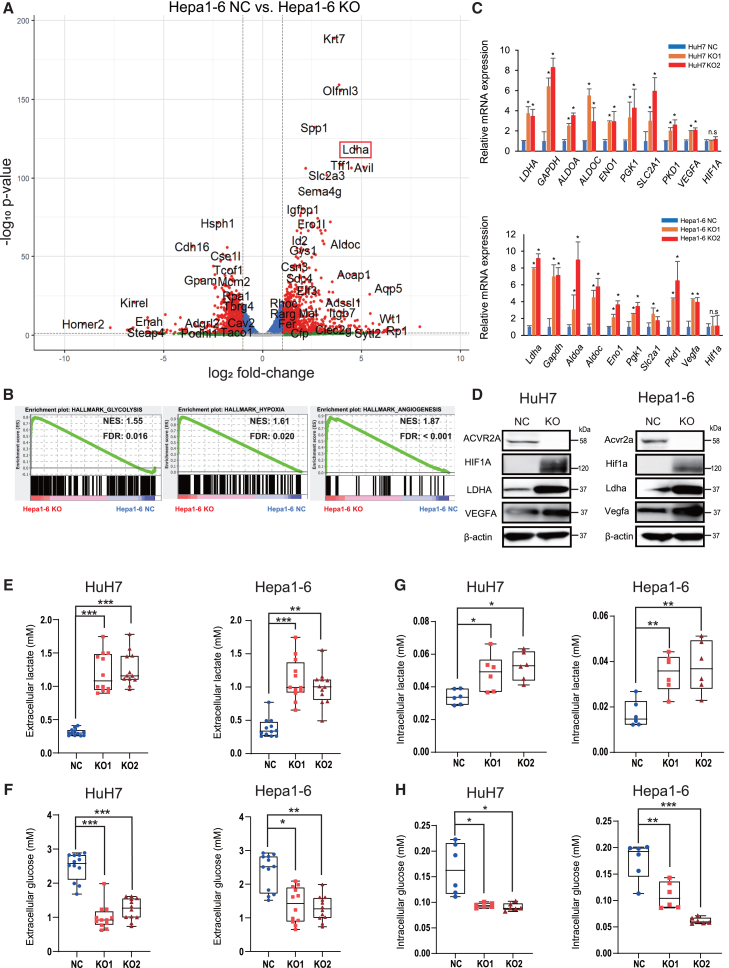
Glycolysis activation and lactate production in *ACVR2A*-KO HCC (A) Volcano plot of genes differentially expressed between Hepa1-6 NC and KO cells. Genes with |log2 fold-change| > 1 and *p* value < 0.05 were highlighted in red. (B) Enrichment plots of gene sets positively associated with *Acvr2a* knockout in Hepa1-6 cells. NES, normalized enrichment score; FDR, false discovery rate. (C) Quantitative RT-PCR analysis of genes upregulated in HuH7 KO and Hepa1-6 KO cells. Bars represent relative mRNA levels compared to the NC cells. The *p* value was calculated by ANOVA with Tukey-Kramer *post hoc* test. (D) Western blot analysis of genes associated with hypoxia and glycolysis. β-Actin was used as an internal control. (E and F) Extracellular lactate (E) and glucose (F) levels. The *p* value was calculated by Kruskal-Wallis test with Steel-Dwass *post hoc* test. (G and H) Intracellular lactate (G) and glucose (H) levels. The *p* value was calculated by Mann-Whitney *U* test. Boxes represent the 25th, 50th, and 75th percentiles. Data are the mean ± SD. n.s, not significant; ∗*p* < 0.05, ∗∗*p* < 0.01, ∗∗∗*p* < 0.001.

RNA-seq analysis was conducted to explore genes differentially expressed between the Hepa1-6 NC and KO cells (Figure 2A and Table S2). Gene set enrichment analysis revealed that *ACVR2A* KO was significantly associated with enhanced glycolysis, hypoxic conditions, and angiogenesis (Figure 2B). Additionally, we conducted gene ontology (GO) enrichment analysis on the differentially expressed genes identified in Figure 2A (log2 fold change > 1 and the top 200 lowest *p* values) and clarified significant enrichment of glycolysis-related biological processes (Figure S3), such as “glycolytic process” (GO:0006096) and “canonical glycolysis” (GO:0061621). Quantitative reverse-transcription PCR (RT-PCR) analysis demonstrated the upregulated expression levels of genes involved in the three biological processes, such as *LDHA*, *GAPDH, ALDOA, ALDOC*, *ENO1*, *PGK1*, *SLC2A1*, *PDK1*, and *VEGFA*, in the KO subclones of HuH7, Hepa1-6, and 3H3-Pten-KO cells (Figures 2C and S4A). Although the mRNA expression level of *HIF1A*, a key regulator of the hypoxic response, was not changed between the NC and KO cells, not only HIF1α but also LDHA and VEGFA were overexpressed in the KO cells at the protein level (Figures 2D and S4B), consistent with the results from the gene set enrichment analysis (Figure 2B). We measured the concentration of lactate and glucose to assess metabolic alterations by *ACVR2A* KO. As expected, the concentration of extracellular and intracellular lactate was higher in the KO cells than in the NC cells, while the concentration of extracellular and intracellular glucose was decreased in the KO cells compared to the NC cells (Figures 2E–2H and S4C–S4F).

### ACVR2A inactivation organizes immunosuppressive tumor microenvironment with high lactate levels

**Figure 3:**
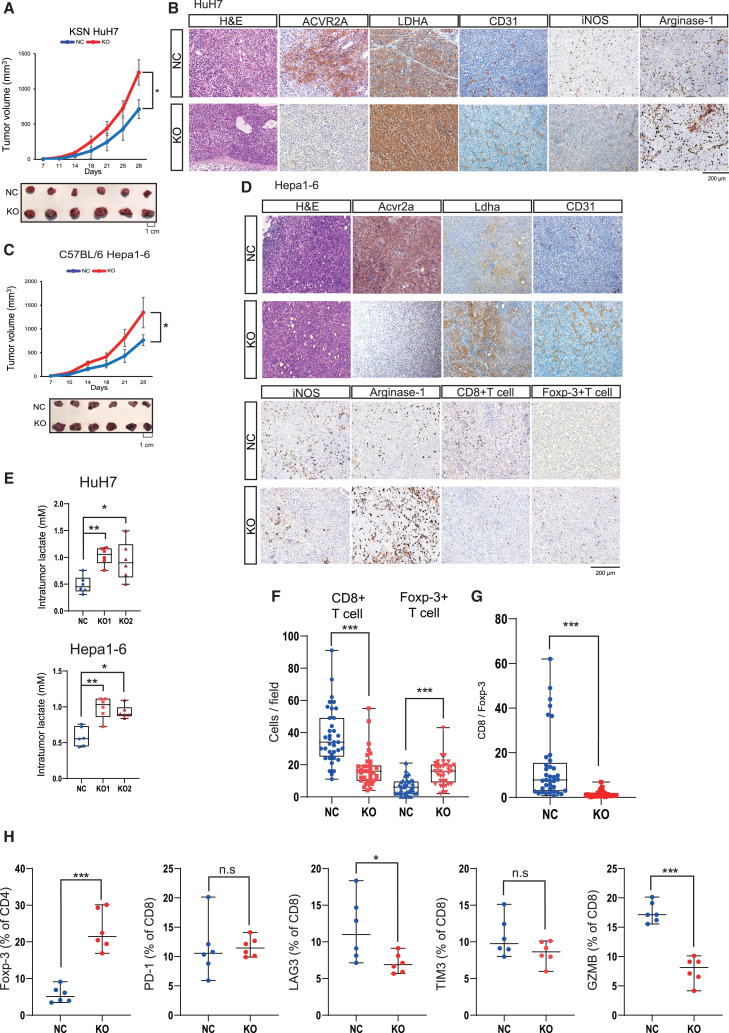
Recruitment of Treg cells under high-lactate conditions of *ACVR2A*-KO HCC (A) Tumorigenicity assay of HuH7 KO cells in immunodeficient mice (*n* = 6). Representative photo images of tumor specimens were included. The *p* value was calculated by Welch’s t test. (B) Representative immunohistochemical images of ACVR2A, LDHA, and endothelial and immune cell markers in tumors derived from HuH7 KO cells. Nuclei were stained with hematoxylin. The scale bar represents 200 μm (C) Tumorigenicity assay of Hepa1-6 KO cells in immunoproficient mice (*n* = 6). Representative photo images of tumor specimens were included. The *p* value was calculated by Welch’s t test. (D) Representative immunohistochemical images of ACVR2A, LDHA, and endothelial and immune cell markers in tumors derived from Hepa1-6 KO cells. The scale bar represents 200 μm (E) Intratumoral lactate levels in HuH7 KO and Hepa1-6 KO cells. The *p* value was calculated using Mann-Whitney *U* test. (F) Quantitative immunohistochemical analysis of CD8^+^ T cell and Foxp-3^+^ Treg cell infiltration. The *p* value was calculated using Mann-Whitney *U* test. (G) CD8^+^ T cell/Foxp-3^+^ Treg cell ratio. The *p* value was calculated using Mann-Whitney *U* test. (H) Quantitative flow cytometric analysis of Foxp-3^+^ Treg cells and CD8^+^ T cells with exhaustion and activation markers. The *p* value was calculated using Mann-Whitney *U* test. H&E, hematoxylin and eosin. Boxes represent the 25th, 50th, and 75th percentiles. Data are the mean ± SD. ∗*p* < 0.05, ∗∗*p* < 0.01, ∗∗∗*p* < 0.001.

We proceeded to evaluate the tumorigenic property and tumor immune microenvironment of *ACVR2A*-KO cells. Upon subcutaneous injection into immunodeficient mice, the HuH7 KO and Hepa1-6 KO cells showed a significant increase in tumor size compared to the NC cells (Figures 3A and S5A). In the grafted tumors, the HuH7 KO cells exhibited depleted expression of ACVR2A at the cell membrane and elevated expression of LDHA at the cytoplasm, and CD31-positive endothelial cells were increasingly recruited (Figure 3B), which indicated the activation of glycolysis and angiogenesis signaling pathways in the *ACVR2A*-KO cells. The concentration of intratumoral lactate was upregulated in the HuH7 and Hepa1-6 KO samples (Figures 3E and S5B). Histopathological evaluation of immune cells revealed a decrease in iNOS-positive M1 macrophages and an increase in arginase-1-positive M2 macrophages. These observations were consistently replicated in the syngeneic subcutaneous transplantation model of Hepa1-6 KO cells (Figures 3C and 3D). Moreover, the Hepa1-6 KO tumor tissues exhibited mild intratumoral infiltration of CD8^+^ T cells and marked enrichment of forkhead box protein 3 (Foxp-3)^+^ Treg cells compared to the Hepa1-6 NC tumor tissues in the syngeneic subcutaneous transplantation model (Figure 3F), indicating the downregulation of the CD8/Foxp-3 ratio (Figure 3G). Flow cytometric analysis of tumor-infiltrating lymphocytes confirmed these immunological features, demonstrating CD8^+^ T cell inactivation and Foxp-3^+^ Treg cell induction (Figures 3H and S6). Treatment with anti-CD25 antibody, frequently used to deplete Treg cells in mice,^22^^,^^23^ significantly inhibited tumor growth of the Hepa1-6 KO cells without affecting body weight or intratumoral lactate levels (Figures S7A–S7C). In the tumor tissues of the Hepa1-6 KO cells, the number of Foxp-3^+^ Treg cells decreased, while the number of granzyme B (GZMB)^+^ T cells increased without upregulating exhaustion markers such as PD-1 and TIM-3 (Figures S7D–S7G). These data are consistent with recent findings that high-lactate and low-glucose conditions hinder CD8^+^ T cell infiltration and promote Foxp-3^+^ Treg cell accumulation.^21^ We also revealed the biological effects of *Acvr2a* KO on the tumorigenic ability, tumor immune microenvironment, and intratumoral lactate levels using 3H3-Pten-KO cells (Figures S4G–S4J).

Furthermore, we employed an orthotopic transplantation model of Hepa1-6 KO cells (Figures S8A–S8C). The Hepa1-6 KO group harbored higher total liver weight and overall tumor weight than the Hepa1-6 NC group (Figure S8D). Similarly to the subcutaneous transplantation model, the tumor specimens generated from the KO cells showed upregulated LDHA expression, decreased CD8^+^ T cell infiltration, and increased Foxp-3^+^ Treg cell infiltration (Figures S8E–S8G). The lactate concentration in the KO tumor tissues was significantly higher compared to the NC tumor tissues as well as normal liver tissues (Figure S8H).

### Histopathological assessment of ACVR2A-low HCC

**Figure 4:**
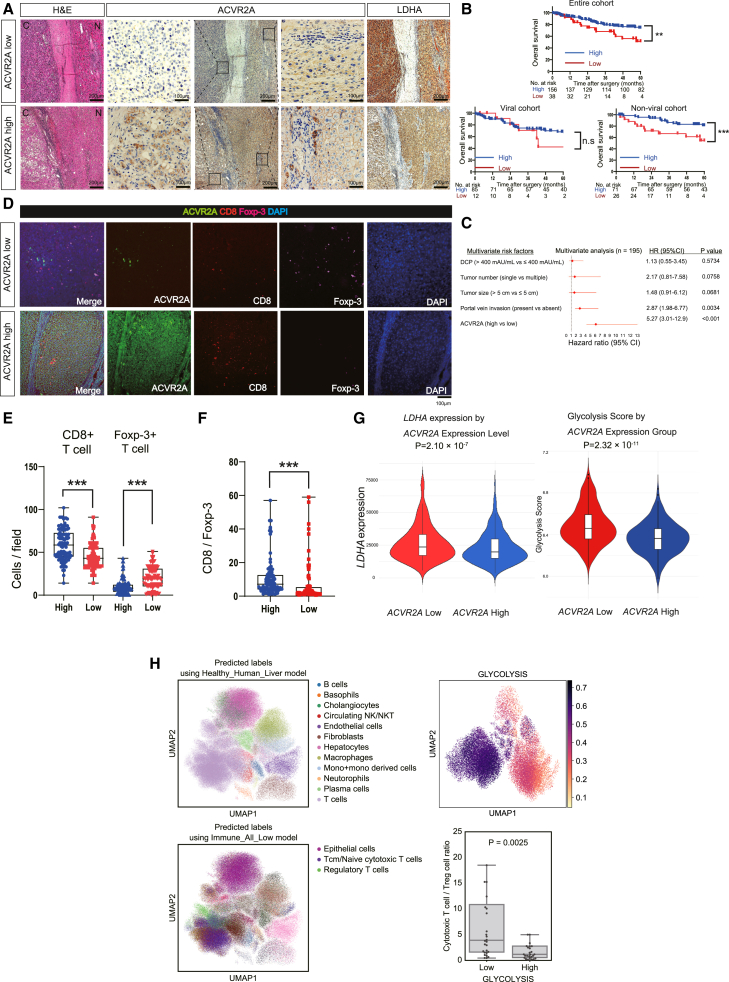
Immunohistopathological evaluation of ACVR2A attenuation, LDHA expression, and Treg cell infiltration in human HCC (A) Representative immunohistochemical images of ACVR2A and LDHA in human HCC samples, indicating the representative ACVR2A-high and -low cases. Nuclei were stained using hematoxylin. H&E, hematoxylin and eosin; C, cancerous tissues; N, adjacent liver tissues. The scale bar represents 100 or 200 μm. (B) Kaplan-Meier curves of OS in patients with the ACVR2A-high and -low HCC groups. The *p* value was calculated using the log rank test. (C) Multivariate analysis of clinicopathological factors associated with OS in the TMDU cohort. HR, hazard ratio; CI, confidence interval. (D) Representative immunofluorescent images of ACVR2A, CD8, and Foxp-3. Nuclei were stained using DAPI. The scale bar represents 100 μm (E) Quantitative analysis of CD8^+^ T cell and Foxp-3^+^ T cell infiltration. The *p* value was calculated using Kruskal-Wallis test with Steel-Dwass *post hoc* test. (F) CD8^+^ T cell/Foxp-3^+^ Treg cell ratio. The *p* value was calculated using Mann-Whitney *U* test. (G) Expression analysis of *LDHA* (left) and enrichment analysis of the glycolytic pathway (right) in the *ACVR2A*-high and -low HCC groups using the TCGA dataset. The scale bar represents 200 μm. (H) Single-cell analysis of HCC samples. The left panels show UMAP plots of 185,469 live cells in six scRNA-seq datasets annotated by the CellTypist program with the healthy liver (upper) and immune cell (lower) models. The upper right panel shows UMAP plot of 31,794 hepatocytes with enrichment scores for the glycolytic pathway estimated by the ssGSEA program. The lower left panel presents the cytotoxic T cell/Treg cell ratio in the glycolysis-high and -low HCC groups (*N* = 28 and 28, respectively). Boxes represent the 25th, 50th, and 75th percentiles. Data are the mean ± SD. n.s, not significant; ∗*p* < 0.05, ∗∗*p* < 0.01, ∗∗∗*p* < 0.001.

To validate the results from both *in vitro* and *in vivo* experiments of *ACVR2A*-KO cells, immunohistochemical analysis was performed using clinical samples of human HCC. Figure 4A demonstrates the normal-tumor border region of each two ACVR2A-high and -low representative cases. Clinicopathological evaluation clarified that the ACVR2A-low group was closely connected to metabolic dysfunction-associated steatotic liver disease/metabolic dysfunction-associated steatohepatitis (MASLD/MASH) as well as high alcohol consumption and no viral infection, consistent with the results from the gene mutation and mRNA expression analyses of the TCGA dataset (Figures 1A–1C and Table S3). In the entire cohort, the ACVR2A-low group displayed unfavorable OS (Figure 4B). The ACVR2A-low group showed worse OS than the ACVR2A-high group in the non-viral cohort, while there was no significant difference in OS between the two groups in the viral cohort. Moreover, univariate Cox regression analysis elucidated that des-γ-carboxy prothrombin, tumor number, tumor size, portal vein invasion, and ACVR2A expression were importantly correlated with OS, and multivariate analysis further identified tumor number and ACVR2A inactivation as independent predictive factors for the prognosis of patients with HCC in the entire cohort (Figure 4C). Immunohistochemical staining also revealed that ACVR2A-low HCC cells exhibited enhanced expression of LDHA, decreased number of CD8^+^ T cells, and increased number of Foxp-3^+^ Treg cells (Figures 4E and 4F). These data were consistent with the results from multiple fluorescent immunostaining (Figure 4D).

To further clarify the relationship between ACVR2A deficiency, hyperglycolysis, and Treg cell infiltration, we conducted comprehensive molecular and immunological analyses using publicly available datasets. We divided HCC samples into *ACVR2A*-high and -low groups using the TCGA transcriptome datasets and discovered that the *ACVR2A*-low group was significantly associated with the high expression level of *LDHA* and the high enrichment score of the glycolytic pathway (Figure 4G). We next integrated six single-cell RNA-seq (scRNA-seq) datasets using the Harmony program and labeled each cell using the CellTypist program (Figure 4H). UMAP plot indicated successfully identified clusters composed of epithelial cells and T cells. We extracted “hepatocytes,” corresponding to HCC cells, and generated UMAP plot of them showing three distinct subclusters, the left of which was correlated with hyperglycolysis. We stratified the HCC samples into glycolysis-high and -low groups and identified that cytotoxic T cell/Treg cell ratio was significantly lower in the glycolysis-high group than in the glycolysis-low group, consistent with our result from immunohistochemical analysis (Figure 3G). Thus, we identified the ACVR2A deficiency-hyperglycolysis-Treg cell infiltration axis from cell biological, pathological, and bioinformatics analyses.

### LDHA expression is repressed by the activin/SMAD signaling pathway

**Figure 5:**
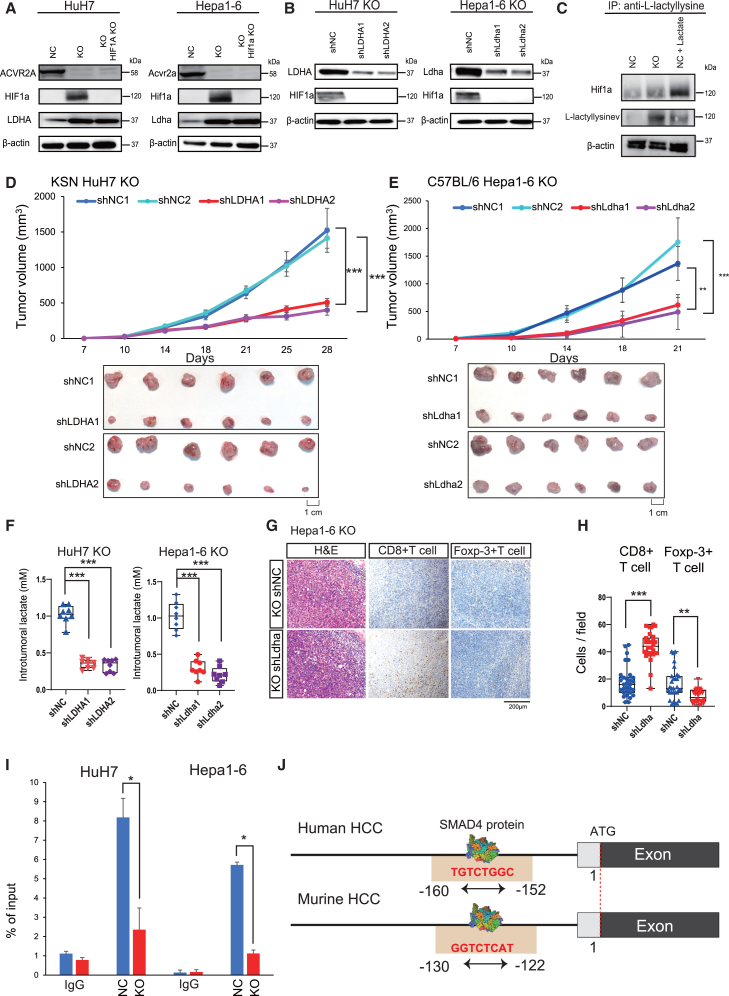
Lactate production induced by LDHA overexpression via the activin/SMAD signaling pathway in *ACVR2A*-KO HCC (A and B) Western blot analysis of HIF1A and LDHA expression in HuH7 KO and Hepa1-6 cells with HIF1A knockout (A) and LDHA knockdown (B). β-Actin was used as an internal control. (C) Co-immunoprecipitation analysis of Hif1a and L-lactyl-lysine. (D and E) Tumorigenicity assay of HuH7 KO cells with *LDHA* knockdown in immunodeficient (D) and immunoproficient (E) mice (*n* = 6). Representative photo images of tumor specimens were included. The *p* value was calculated using Kruskal-Wallis test with Steel-Dwass *post hoc* test. (F) Intratumoral lactate levels in tumors derived from Hepa1-6 KO cells with *Ldha* knockdown. The *p* value was calculated using Kruskal-Wallis test with Steel-Dwass *post hoc* test. (G) Representative immunohistochemical images of CD8^+^ T cells and Foxp-3^+^ T cells. Nuclei were stained with hematoxylin. (H) Quantitative analysis of CD8^+^ T cell and Foxp-3^+^ T cell infiltration. The *p* value was calculated using Mann-Whitney *U* test. (I) Quantitative ChIP analysis of SMAD4 at the promoter region of *LDHA*. The *p* value was calculated by Welch’s t test. (J) Schematic representation of the promoter region of *LDHA*. H&E, hematoxylin and eosin. Boxes represent the 25th, 50th, and 75th percentiles. Data are the mean ± SD. ∗*p* < 0.05, ∗∗*p* < 0.01, ∗∗∗*p* < 0.001.

We aimed to clarify the molecular mechanism underlying the activation of glycolysis, hypoxia, and angiogenesis signaling pathways, which play essential functions in cancer progression and immune evasion in ACVR2A-deficient cells. Surprisingly, when *HIF1A* KO was introduced into *ACVR2A*-KO HuH7 cells, *LDHA* expression exhibited minimal changes (Figure 5A). The similar finding was also obtained in Hepa1-6 cells with double KO of *Acvr2a* and *Hif1a* (Figure 5A), suggesting a HIF1α-independent molecular mechanism. *Hif1a* KO abrogated the proliferation ability of Hepa1-6 NC cells, but not Hepa1-6 KO cells (Figure S9A). Subcutaneous transplantation into C57BL/6 mice yielded no important difference in tumor size between *Acvr2a*-KO and *Acvr2a*/*Hif1a*-KO Hepa1-6 cells (Figure S9B).

We next utilized short hairpin RNA (shRNA) targeting *LDHA* in *ACVR2A*-KO cells (Figure S10A), resulting in the downregulation of HIF1α protein (Figure 5B). Considering recent studies demonstrating that metabolites like lactate, succinate, and succinylacetone can stabilize HIF1α under normoxic conditions^24^^,^^25^^,^^26^ and a previous study reporting that HIF1α lactylation at lysine residues contributes to the protein’s stabilization,^27^ we conducted immunoprecipitation analysis of HIF1α and lactyl-lysine in ACVR2A-KO HCC cells. HIF1α was strongly expressed in Hepa1-6 cells with *Acvr2a* KO and lactate treatment compared to the control, and the lactylation level of HIF1α was notably increased in *Acvr2a*-KO cells (Figure 5C). *LDHA* KD diminished the cell proliferation and colony formation ability (Figures S10B and S10C) and reduced the concentration of extracellular and intracellular lactate (Figures S10D and S10E) in both HuH7 and Hepa1-6 KO cells. Tumor growth was inhibited by *LDHA* KD in the subcutaneous transplantation model of HuH7 and Hepa1-6 KO cells (Figure 5DE). Expectedly, *LDHA* KD downregulated the intratumoral lactate levels in both human and mouse transplanted tumor samples (Figure 5F). In the tumor tissues of Hepa1-6 cells with *Acvr2a* KO and *Ldha* KD, the number of infiltrating CD8^+^ T cells was increased, while the number of Foxp-3^+^ Treg cells was decreased (Figure 5GH).

To test whether the SMAD signal transduction was altered by *ACVR2A* KO, both the NC and KO cells were exposed to activin, followed by the collection of the cells at 1.5, 3, and 6 h intervals. SMAD2 and SMAD3 phosphorylation was time-dependently increased in the NC cells, although no phosphorylation was observed in the KO cells (Figures S10F and S10G). Furthermore, western blot analysis showed that nuclear translocation of SMAD4 was inhibited in the KO cells (Figure S10H). We employed JASPAR to detect the binding sites of SMAD4 and conducted chromatin immunoprecipitation (ChIP) analysis of SMAD4 at the promoter of *LDHA*, which demonstrated that SMAD4 protein more frequently resided at the predicted binding site in the NC cells than in the KO cells (Figures 5I and 5J).

### MCT inhibition downregulates the concentration of intratumoral lactate and subsequently attenuates the induction of Treg cells

**Figure 6:**
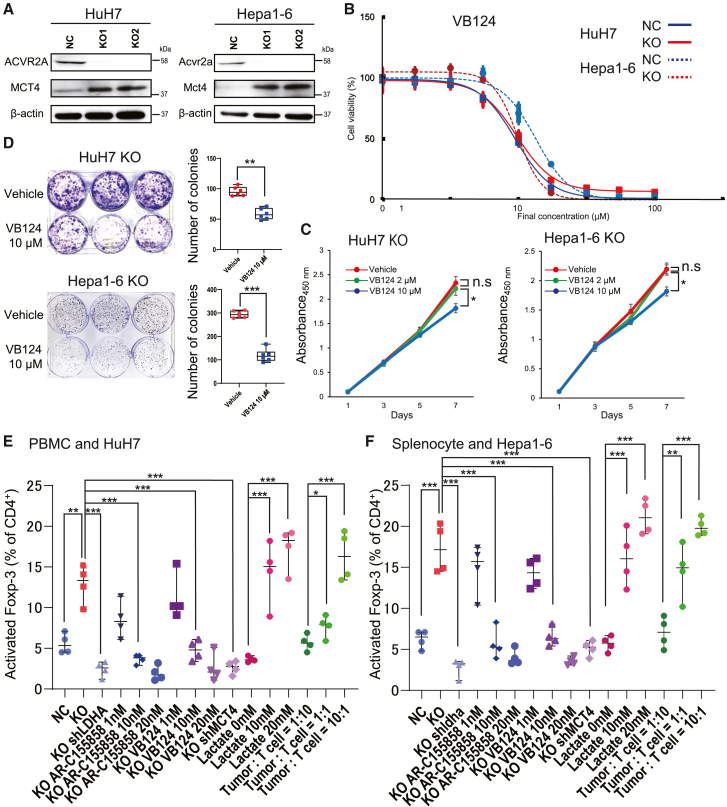
Impairment of Treg cell infiltration by MCT inhibition in *ACVR2A*-KO HCC (A) Western blot analysis of MCT4 expression in HuH7 KO and Hepa1-6 KO cells. β-Actin was used as an internal control. (B) Dose-response curves of the MCT4 inhibitor VB124 in HuH7 KO and Hepa1-6 KO cells. (C and D) Proliferation (C) and colony formation (D) assays of HuH7 KO and Hepa1-6 KO cells treated with VB124. Representative photo images in each assay were included. The *p* value was calculated by Welch’s t test (C). The *p* value was calculated by Mann-Whitney *U* test (D). (E and F) Quantitative flow cytometric analysis of Foxp-3^+^ Treg cells co-cultured with HuH7 KO cells (E) and Hepa1-6 KO cells (F). The *p* value was calculated using Mann-Whitney *U* test or Kruskal-Wallis test with Steel-Dwass *post hoc* test. Boxes represent the 25th, 50th, and 75th percentiles. Data are the mean ± SD. n.s, not significant; ∗*p* < 0.05, ∗∗*p* < 0.01, ∗∗∗*p* < 0.001.

To investigate the relationship between the high-lactate milieu and Treg cell infiltration, we tried pharmacological regulation of lactate transporters including MCT1 and MCT4. We first performed western blot analysis of MCT4 and observed the upregulation of MCT4 expression in *ACVR2A*-KO subclones of both HuH7 and Hepa1-6 cells (Figure 6A). The half-minimal inhibitory concentration (IC50) of the MCT4 inhibitor VB124 was approximately 10 μM in both the NC and KO cells (Figure 6B). Treatment with 10 μM VB124 abrogated the proliferation and colony formation activity in HuH7 KO and Hepa1-6 KO cells (Figures 6C and 6D). While the extracellular lactate concentration was decreased under VB124 administration, the intracellular lactate concentration remained unchanged (Figures S11A and S11B).

We conducted a co-culture of HuH7 cells and peripheral blood mononuclear cells (PBMCs) and assessed the ratio of Foxp-3^+^ Treg cells to CD4^+^ T cells (Figure S12A). The proportion of Foxp-3^+^ Treg cells rose from 5% to 20% during co-culture with the NC cells and KO cells. KD of *LDHA* and treatment with the MCT1 inhibitor AR-C155858 and the MCT4 inhibitor VB124 significantly reduced the population of Foxp-3^+^ Treg cells (Figure 6E). The similar observations were consistently seen in the cases of Hepa1-6 and 3H3-Pten-KO cells (Figures 6F and S4K). The proportion of CD8^+^ T cells were not significantly changed (Figure S12B). These trends were observed in a dose-dependent manner, although the cell viability was often reduced at the highest dose (Figures S12C and S12D).

Overexpression of *Ldha* and *Mct4* in the Hepa1-6 cells increased the concentration of extracellular lactate and the number of Treg cells under co-culture system (Figures S13A–S13C). These cancer cells showed enhanced tumorigenic ability when subcutaneously injected into C57BL/6 mice (Figures S13D and S13E). The concentration of intratumoral lactate and the proportion of Treg cells were markedly elevated in the tumor tissues, while CD8^+^ T cell infiltration was mild, resulting in the downregulation of the CD8/Foxp-3 ratio (Figures S13F–S13J).

We detected the high expression levels of *MCT1* and *MCT3* as lactate transporters other than *MCT4* in HCC tissues using the TCGA dataset (Figure S14A). Although *Mct1*, but not *Mct3*, was predominantly expressed in the Hepa1-6 KO cells (Figure S14B), KD of *Mct1* failed to affect the extracellular lactate concentration and the Foxp-3^+^ Treg cell proportion under co-culture condition, suggesting the essential roles of MCT4 in lactate transporting within HCC cells (Figures S14C–S14E).

### MCT4 inhibition enhances the efficacy of anti-PD-1 antibody therapy

Intraperitoneal injection of anti-PD-1 antibody into mice with tumors derived from Hepa1-6 NC and KO cells was initiated on day 7 with a dosage of 200 μg per head every week (Figure S15A). While PD-1 blockade reduced the tumor size of Hepa1-6 NC cells, there was no evident response to anti-PD-1 therapy in Hepa1-6 KO tumors (Figure S15B). Immunostaining showed that infiltration of both CD8^+^ T cells and Foxp-3^+^ Treg cells was increased in both NC and KO tumors when treated with anti-PD-1 antibody (Figures S15C and S15D). However, administration of anti-PD-1 antibody significantly upregulated the CD8/Foxp-3 ratio in NC tumors, but not in KO tumors (Figure S15E). Considering the immunosuppressive roles of Treg cells, the administration of anti-CTLA-4 antibody into mice with Hepa1-6 KO tumors yielded slight influences on tumor growth (Figure S15F). Although the treated group displayed a substantial increase in CD8^+^ T cell infiltration, the number of Foxp-3^+^ Treg cells and the CD8/Foxp-3 ratio showed no significant difference between the untreated and treated groups (Figures S15G–S15I).

**Figure 7:**
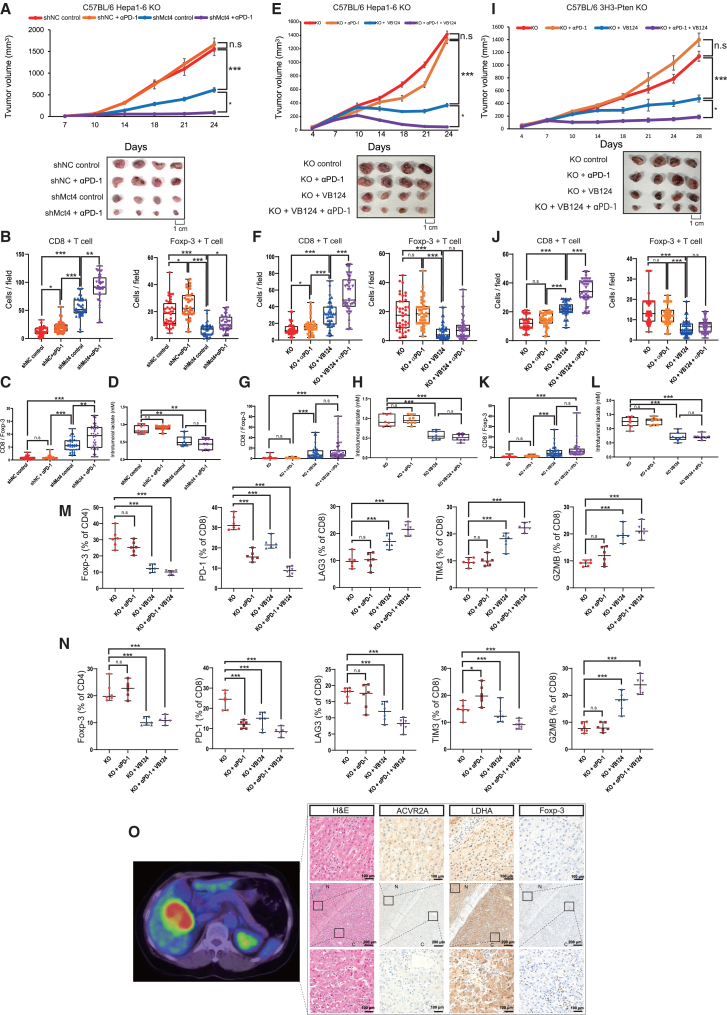
Synergistic anti-tumor effects of MCT inhibition and immune checkpoint blockade on *ACVR2A*-KO HCC (A) Tumorigenicity assay of Hepa1-6 KO cells with *Mct4* knockdown in immunoproficient mice treated with anti-PD-1 antibody (*n* = 4). Representative photo images of tumor specimens were included. The *p* value was calculated by ANOVA with Tukey-Kramer *post hoc* test. (B) Quantitative immunohistochemical analysis of CD8^+^ T cell and Foxp-3^+^ T cell infiltration. The *p* value was calculated using Kruskal-Wallis test with Steel-Dwass *post hoc* test. (C) CD8^+^ T cell/Foxp-3^+^ Treg cell ratio. The *p* value was calculated using Kruskal-Wallis test with Steel-Dwass *post hoc* test. (D) Intratumoral lactate levels. The *p* value was calculated using Kruskal-Wallis test with Steel-Dwass *post hoc* test. (E) Tumorigenicity assay of Hepa1-6 KO cells in immunoproficient mice treated with anti-PD-1 antibody and VB124 (*N* = 4). Representative photo images of tumor specimens were included. The *p* value was calculated by ANOVA with Tukey-Kramer *post hoc* test. (F) Quantitative immunohistochemical analysis of CD8^+^ T cell and Foxp-3^+^ T cell infiltration. The *p* value was calculated using Kruskal-Wallis test with Steel-Dwass *post hoc* test. (G) CD8^+^ T cell/Foxp-3^+^ Treg cell ratio. The *p* value was calculated using Kruskal-Wallis test with Steel-Dwass *post hoc* test. (H) Intratumoral lactate levels. The *p* value was calculated using Kruskal-Wallis test with Steel-Dwass *post hoc* test. (I) Tumorigenicity assay of 3H3-Pten-KO cells with *Acvr2a* knockout in immunoproficient mice treated with anti-PD-1 antibody and VB124 (*N* = 4). Representative photo images of tumor specimens were included. The *p* value was calculated by ANOVA with Tukey-Kramer *post hoc* test. (J) Quantitative immunohistochemical analysis of CD8^+^ T cell and Foxp-3^+^ T cell infiltration. The *p* value was calculated using Kruskal-Wallis test with Steel-Dwass *post hoc* test. (K) CD8^+^ T cell/Foxp-3^+^ Treg cell ratio. The *p* value was calculated using Kruskal-Wallis test with Steel-Dwass *post hoc* test. (L) Intratumoral lactate levels. The *p* value was calculated using Kruskal-Wallis test with Steel-Dwass *post hoc* test. (M and N) Quantitative flow cytometric analysis of Foxp-3^+^ Treg cells and CD8^+^ T cells with exhaustion and activation markers in Hepa1-6 KO (M) and 3H3-Pten-KO with *Acvr2a*-knockout (N) xenografts. The *p* value was calculated using Mann-Whitney *U* test. (O) Representative case of HCC with high SUVmax. The left and right panels show an FDG-PET/CT image and representative immunohistochemical images of ACVR2A, LDHA and Treg cells, respectively. Nuclei were stained using hematoxylin. The scale bar represents 100 or 200 μm. Boxes represent the 25th, 50th, and 75th percentiles. Data are the mean ± SD. n.s, not significant; ∗*p* < 0.05, ∗∗*p* < 0.01, ∗∗∗*p* < 0.001.

To examine the anti-tumor potential of lactate control on *ACVR2A*-KO tumors, we first established HuH7 KO and Hepa1-6 KO cells with tetracycline-inducible sh*MCT4*. Treatment with 30 ng/mL doxycycline downregulated the mRNA and protein levels of MCT4 in both tumor cell lines (Figures S11C and S11D). In the KO cells, the extracellular lactate concentration was diminished by *MCT4* KD, whereas the intracellular lactate concentration was unaltered (Figures S11E and S11F). *MCT4* KD impaired the proliferation and colony formation property only in the KO cells (Figures S11G and S11H). When HuH7 KO and Hepa1-6 KO cells were subcutaneously transplanted into immunodeficient mice, *MCT4* KD failed to exert inhibitory effects on tumor development (Figures S16A and S16B), despite remarkable reduction of the intracellular lactate concentration (Figure S16C). In contrast, upon the syngeneic transplantation model of Hepa1-6 cells, *Mct4* KD successfully suppressed tumor growth and restored the vulnerability to anti-PD-1 therapy (Figure 7A), suggesting that lactate control could affect immune cells rather than tumor cells. Immunohistochemical analysis unveiled an increased number of CD8^+^ T cells and a decreased number of Foxp-3^+^ Treg cells in the *Mct4*-KD group (Figures 7B and S17A). Additionally, treatment with anti-PD-1 antibody promoted CD8^+^ T cell infiltration and enhanced the CD8/Foxp-3 cell ratio in tumor tissues derived from *Mct4*-KD cells (Figure 7C). A marked decrease in the intratumoral lactate concentration was confirmed in the *Mct4*-KD tumor specimens (Figure 7D). For clinical application, we confirmed these findings using the MCT4 inhibitor. VB124 monotherapy exerted suppressive effects on tumor tissues derived from the Hepa1-6 KO cells and 3H3-Pten-KO cells with *Acvr2a* KO, and its combination with anti-PD-1 antibody significantly enhanced the anti-tumor response (Figures 7E–7L). Similar to the genetic KD, pharmacological inhibition of MCT4 successfully upregulated the CD8/Foxp-3 ratio by increasing CD8^+^ T cells and decreasing Treg cells and activated CD8^+^ T cells expressing GZMB without inducing exhaustion markers (Figures 7M, 7N, S17B, and S17C). No significant changes were observed in body weight or the pathological features of organs including the heart, lungs, stomach, liver, spleen, kidneys, intestines, and muscles in mice following VB124 administration (Figures S18A–S18C).

To investigate whether preoperative hyperglycolysis can be predicted by imaging, we immunostained HCC samples resected from patients who underwent 18F-fluorodeoxyglucose positron emission tomography-computed tomography (FDG-PET/CT), and FDG-PET hyperaccumulation significantly correlated with increased LDHA expression and enhanced Treg cell recruitment (*p* = 0.006 and 0.039, respectively) shown in Table S4. A representative ACVR2A-low/LDHA-high/Treg cell-rich case exhibited FDG-PET hyperaccumulation (Figure 7O).

## Discussion

Comprehensive genomic analysis identified more frequent mutations of *ACVR2A* in non-viral HCC than in viral HCC (Figure 1A), consistent with the previous finding that the *ACVR2A* mutation rate is higher in NASH-HCC than in other HCC etiologies.^15^ Two large-scale transcriptomic analyses demonstrated the tight connection between low *ACVR2A* expression and poor patient prognosis specifically in non-viral HCC cases (Figures 1B and 1C). Immunohistochemical assessment of human HCC samples validated these results and moreover revealed that ACVR2A inactivation correlated with Treg cell accumulation (Figure 4D). We have currently addressed that metabolism-associated liver cancer is characterized by Treg cell enrichment,^28^ and Fujita et al. have documented worse OS in Treg subclass than in other subclasses,^29^ which supports our histopathological and immunological observations. In our syngeneic transplantation model of *Acvr2a*-KO HCC, treatment with anti-PD-1 antibody could exhibit no inhibitory impacts on tumor growth. Recent studies have reported that NASH-HCC is refractory to anti-PD-1 therapy,^13^ part of which may be caused by ACVR2A-deficient HCC. Thus, ACVR2A inactivation in non-viral and metabolic disease-related HCC recruits Treg cells, leading to unfavorable outcome and extrinsic resistance to PD-1 blockade.

Although it is known that the activin/SMAD signaling pathway suppresses hepatocyte proliferation,^16^ its roles in hepatocarcinogenesis remain unclear. The SMAD signaling pathway influences various cellular process including cell growth, differentiation, and development,^30^^,^^31^^,^^32^ and two recent studies have reported its functions in glucose metabolism of cancer cells. In pancreatic ductal adenocarcinoma, loss of SMAD4 induces the upregulation and nuclear translocation of the glycolytic enzyme PGK1, resulting in high oxidative phosphorylation and metastatic potential.^20^ SMAD4 deficiency promotes colorectal cancer progression activating aerobic glycolysis through the upregulation of GLUT1 expression.^33^ In addition to these previous findings, we revealed that *ACVR2A* impairment increased the expression levels of genes involved in the glycolysis pathway via the activin/SMAD signal transduction in non-viral HCC (Figures 2A–2D) and identified that SMAD4 repressed LDHA expression by directly binding to the promoter region (Figures 5I and 5J). Taken together, SMAD signal inactivation may contribute to cancer progression through the induction of glycolytic processes.

Metabolic reprogramming is a hallmark of malignancy, and cancer cells facilitate glycolytic catabolism of glucose to lactate, known as the Warburg effect.^34^ This biological alteration contributes to glucose depletion and lactate-derived acidification in tumor microenvironment, which causes metabolic competition between cancer cells and immune cells and direct suppression of anti-tumor immune response.^35^ However, Treg cells can tolerate low-glucose and high-lactate conditions by Foxp-3-mediated induction of oxidative phosphorylation and NAD(H) oxidation^36^ and rather require lactate uptake via MCT1.^37^ Treg cells play essential roles in immunosuppression within normal and tumor tissues, and accumulating evidence indicates that Treg cells assist cancer cells to evade immunosurveillance in the liver.^21^ Compared to primary lesions, liver metastatic lesions exhibit enhanced glycolysis along with an increase of Treg cells.^21^^,^^38^^,^^39^ This study clarifies that lactate production through the activin/SMAD/LDHA axis serves as a mechanism for the recruitment of Treg cells in the HCC subtype (Figures 6E and 6F).

Although advance in immunotherapy including immune checkpoint blockade (ICB) has dramatically improved cancer patient survival, several tumor-intrinsic and -extrinsic factors limit the clinical impacts, one of which is intratumoral infiltration of Treg cells.^14^^,^^40^^,^^41^ PD-1 blockade reinvigorates not only cytotoxic T lymphocytes but also Treg cells, which is linked to ICB resistance and even hyperprogression disease during the treatment.^42^^,^^43^ To solve this dilemma, the development of Treg cell-targeted drugs for combination therapy is in progress. Since Treg cells constitutively express CTLA-4, competing with CD28 for the co-stimulatory ligands CD80 and CD86, on the cell surface, anti-CTLA-4 antibody blocks the immunosuppressive function of Treg cells and attacks Treg cells by Fc-mediated antibody-dependent cellular cytotoxicity and cellular phagocytosis.^41^ To date, anti-tumor effects of anti-CTLA-4 therapy are still under debate, and Zappasodi et al. have reported that CTLA-4 blockade derives Treg cell destabilization only under low-glycolytic conditions,^44^ consistent with our finding that anti-CTLA-4 therapy is ineffective for *ACVR2A*-KO HCC (Figure S15F). Given the lactate reliance of Treg cells,^37^ inhibition of lactate influx via MCT1 is a therapeutic strategy that targets Treg cells.^45^ The MCT1 inhibitor AZD3965 has completed the phase 1 dose-escalation trial^46^ and is greatly anticipated for future clinical applications. In the present study, *ACVR2A*-KO HCC tissues were enriched with Treg cells and insusceptible to anti-PD-1 therapy (Figures S15B–S15E), and the combination with the MCT4 inhibitor VB124 could ameliorate the CD8/Foxp-3 ratio and attenuate tumor growth (Figures 7E–7L). These data suggest that our syngeneic mouse model is a valuable tool for basic research on biological and molecular mechanisms and preclinical evaluation of therapeutic strategies for *ACVR2A*-deficient and Treg cell-rich HCC.

To diagnose HCC with high glycolytic activity and aggressive Treg cell infiltration before treatment, FDG-PET/CT imaging may be useful. Borm et al. have reported that FDG uptake in lung cancer in nivolumab treatment is positively associated with Treg cell infiltration in the regional lymph nodes and inversely associated with OS.^47^ Furthermore, high maximum standard uptake value (SUVmax) on FDG-PET/CT significantly correlates with Treg cell recruitment in both gastric and lung cancers.^48^^,^^49^ Consistent with the two previous papers, this study demonstrated FDG accumulation on PET/CT indicating increased LDHA expression and Treg cell infiltration (Figure 7O). Thus, FDG-PET/CT has the potential as an imaging biomarker to predict the efficacy of anti-PD-1 monotherapy and combination therapy of MCT inhibitor and anti-PD-1 antibody. Unfortunately, our hypothesis could not be validated due to the insufficient number of patients undergoing FDG-PET/CT scans, and further studies are needed.

In conclusion, we conducted a comprehensive investigation into the underlying mechanism that ACVR2A inactivation, which is prevalent in non-viral HCC, induces lactate production and secretion through the activin/SMAD/LDHA axis using syngeneic mouse models. Furthermore, these findings are validated through examination of human clinical specimens. High-lactate milieu fosters Treg cell-mediated immune evasion and resistance to conventional immunotherapeutic approaches, which could be resolved by the combination with MCT inhibition.

### Limitations of the study

Despite the significant findings, this study has several limitations. First, MCT4 inhibitors have not yet been tested in clinical trials, leaving their efficacy and possible side effects in patients unknown. However, the promising results from a phase 1 clinical trial of the MCT1 inhibitor AZD3965 suggest that MCT4 inhibitors may hold therapeutic potential. Second, the small sample size of FDG-PET cases may limit the generalizability of our findings. This limitation is further compounded by the single-center design of the study, which raises concerns about selection bias and institutional variations in imaging protocols or patient characteristics. Lastly, transcriptomic datasets and scRNA-seq have inherent technical limitations in capturing the tumor microenvironment of ACVR2A-deficient HCC. This underscores the need for further analyses using spatial transcriptomics and metabolic flux analysis to gain a more comprehensive understanding.

## Resource availability

### Lead contact

Further information and requests for resources and reagents should be directed to and will be fulfilled by the lead contact, Shinji Tanaka (tanaka.monc@tmd.ac.jp).

### Materials availability

This study did not generate new unique reagents. The plasmids used in this study were modified from those purchased from Addgene (Watertown, MA, USA) and VectorBuilder (Chicago, IL, USA), and therefore their redistribution is strictly prohibited. For additional information and inquiries regarding resources and reagents, interested parties are encouraged to contact the lead contact, who will handle and fulfill these requests.

### Data and code availability

• RNA-seq data have been deposited at GEO with the accession number GEO: GSE248922 and are publicly available as of the date of publication.
• This study did not generate new original code.
• Any additional information required to reanalyze the data reported in this paper is available from the lead contact upon request.

## Acknowledgments

We greatly thank Ms Hiromi Nagasaki and Ms Hiromi Onari for technical and clerical assistance. Plasmids for CRISPR-Cas9-mediated genome engineering (lentiCRISPR v2, lentiGuide-Puro, and lentiCas9-Blast) and lentiviral infection (pCMVΔR8.2 and pHCMV-VSV-G) were gifted from Dr. Feng Zhang and Dr. Irvin Chen, respectively.

This study was supported by Grants-in-Aid for Scientific Research (A, 19H01055; B, 23H02979; and C, 22K08864) and Challenging Research (Exploratory, 20K21627 and 22K19554) from the Ministry of Education, Culture, Sports, Science and Technology of Japan (MEXT); P-CREATE (JP19cm0106540) and Program for Basic and Clinical Research on Hepatitis (JP23fk0210102, JP23fk0210090, JP23fk0210106, and JP23fk0210136) from 10.13039/100009619Japan Agency for Medical Research and Development (AMED); and Research Grant from the Princess Takamatsu Cancer Research Fund (JP23fk0210090, JP24fk0210102, JP24fk0210106, JP24fk0210136, and JP24fk0210149).

## Author contributions

K.Y., S.S., and S. Tanaka designed the project and wrote the manuscript. K.Y., S.S., Y.A., and T.T. performed cell biological, histopathological, and bioinformatics analysis. K.Y., T.T., Y.I., S. Tsukihara, Y.T., K.U., A.N., M.Y., A.K., K.A., and M.T. contributed to data curation. Y.A., A.S., Y.S., and M.T. helped write, review, and edit the manuscript. S. Tanaka was responsible for the overall content of this study.

## Declaration of interests

The authors declare no competing interests.

## STAR★Methods

### Key resources table

**Table:** 

REAGENT or RESOURCE	SOURCE	IDENTIFIER
**Antibodies**		

Zombie Aqua™ Fixable Viability Kit	Biolegend	Cat# 423101
7-AAD Viability Staining Solution	Biolegend	Cat# 420404; RRID: AB_2869266
FITC anti-human CD4 Antibody	Biolegend	Cat# 300506; RRID: AB_314074
Anti-human FOXP3-PE (236A/E7)	eBioscience	Cat# 12-4777-82; RRID: AB_1944444
Anti-mouse CD4-V500 (RM4-5)	BD Biosciences	Cat# 560782; RRID: AB_1937315
Anti-mouse FOXP3-PE (FJK-16s)	eBioscience	Cat# 12-5773-82; RRID: AB_465936
BD Pharmingen™ FITC Mouse Anti-Human CD45RA	BD Biosciences	Cat# 555488; RRID:AB_395879
Goat anti-Mouse IgG (H + L) Cross-Adsorbed Secondary Antibody, Alexa Fluor™ 647	Thermo Fisher Scientific	Cat# A-21235; RRID:AB_2535804
Goat anti-Mouse IgG (H + L) Cross-Adsorbed Secondary Antibody, Alexa Fluor™ 568	Thermo Fisher Scientific	Cat# A-11004; RRID:AB_2534072
ACVR2A Antibody - N-terminal region	Aviva systems bilogy	Cat# OAAB16873
LDHA-Specific Polyclonal antibody	Proteintech	Cat# 19987-1-AP; RRID:AB_10646429
CD31 (PECAM-1) (89C2) Mouse mAb	Cell Signaling Technology	Cat# 3528; RRID:AB_2160882
Anti-iNOS antibody (ab15323)	abcam	Cat# ab15323; RRID:AB_301857
Arginase-1 (D4E3M™) XP® Rabbit mAb	Cell Signaling Technology	Cat# 93668; RRID:AB_2800207
CD8α (C8/144B) Mouse mAb	Cell Signaling Technology	Cat# 70306; RRID:AB_2799781
BD Pharmingen™ Purified Mouse anti-Human FoxP3	BD Biosciences	Cat# 560044; RRID:AB_1645589
MCT4 Polyclonal Antibody	Proteintech	Cat# 36169S; RRID:AB_2799095
PGI 20139-1-AP MCT1 Polyclonal antibody	Proteintech	Cat# 20139-1-AP; RRID:AB_2878645
HIF-1α (D1S7W) XP® Rabbit mAb	Cell Signaling Technology	Cat# 22787-1-AP; RRID:AB_11182479
Anti-VEGFA antibody	abcam	Cat# ab46154; RRID:AB_2212642
Phospho-SMAD2 (Ser465/467) (138D4) Rabbit mAb	Cell Signaling Technology	Cat# 3108; RRID:AB_490941
Smad2 (D43B4) XP® Rabbit mAb	Cell Signaling Technology	Cat# 3108; RRID:AB_10626777
Phospho-SMAD3 (Ser423/425) (C25A9) Rabbit mAb	Cell Signaling Technology	Cat# 9520; RRID:AB_2193207
SMAD3 (C67H9) Rabbit mAb #9523	Cell Signaling Technology	Cat# 9523; RRID:AB_2193182
SMAD4 (D3R4N) XP® Rabbit mAb	Cell Signaling Technology	Cat# 46535; RRID:AB_2736998
β-Actin (13E5) Rabbit mAb	Cell Signaling Technology	Cat# 4970; RRID:AB_2223172
Anti-CD31 antibody	abcam	Cat# ab28364; RRID:AB_726362
CD8α (D4W2Z) XP® Rabbit mAb	Cell Signaling Technology	Cat# 98941; RRID:AB_2756376
FoxP3 (D6O8R) Rabbit mAb	Cell Signaling Technology	Cat# 12653; RRID:AB_2797979
BD Pharmingen™ PE Rat Anti-Mouse CD45RA	BD Biosciences	Cat# 553380; RRID:AB_394822
BD OptiBuild™ BV650 Rat Anti-Mouse CD279 (PD-1)	BD Biosciences	Cat# 748266; RRID:AB_2872694
PE anti-mouse CD223 (LAG-3) Antibody	Biolegend	Cat# 125207; RRID:AB_2133344
CD366 (TIM3) Monoclonal Antibody (RMT3-23), PE, eBioscience™	ThermoFisher	Cat# 12-5870-81; RRID:AB_465973
Granzyme B Monoclonal Antibody (NGZB), PE, eBioscience™	ThermoFisher	Cat# 12-8898-80; RRID:AB_10853811

**Biological samples**		

healthy donor PBMC	CTL	N/A

**Chemicals, peptides, and recombinant proteins**		

Fetal Bovine Serum	Biosera	Cat# FB-1061/500
D-MEM (High Glucose) with L-Glutamine and Phenol Red	Fujifilm Wako	Cat# 044-29765
RPMI-1640 with L-Glutamine and Phenol Red	Fujifilm Wako	Cat# 189-02025
RPMI 1640 Medium (ATCC modification)	ThermoFisher	Cat# A1049101
AIM V™ Medium, liquid (research grade)	ThermoFisher	Cat# 12055083
Plus Human Serum AB Xeno-Free-Item	GemCell	Cat# 100-912
Lipofectamine™ 2000 Transfection Reagent	Invitrogen	Cat# 11668027
Puromycin	ThermoFisher	Cat# A1113802
Hygromycin	Fujifilm Wako	Cat# 084-07681
Blasticidin S Hydrochloride	Fujifilm Wako	Cat# 3513-03-9
AR-C155858	ChemScene LLC	Cat# CS-0540
VB124	MedChemExpress	Cat# HY-139665
InVivoMAb anti-mouse PD-1 (CD279)	Bioxcell	Cat# BE0033-2
InVivoMAb anti-mouse CTLA-4 (CD152)	Bioxcell	Cat# BE0131
InVivoPlus anti-mouse CD25 (IL-2Rα)	Bioxcell	Cat# BP0012
Crystal Violet	Fujifilm Wako	Cat# 031–04852 PGI
Doxycycline Hyclate	Sigma-Aldrich	Cat# D4116
recombinant human/mouse/rat activin A PLUS protein	MBL	Cat# QK005-0100
CD3 Monoclonal Antibody (OKT3), Functional Grade	eBioscience	Cat# 16-0037-85
CD28 Monoclonal Antibody (CD28.2), Functional Grade	eBioscience	Cat# 16-0289-85
IL2 (Interleukin-2) Recombinant Human Protein	ThermoFisher	Cat# CTP0021
IL-7, Murine, Recombinant	PeproTech	Cat# 217-17-10UG
IL-15, Murine, Recombinant	PeproTech	Cat# 210-15-10UG
Matrigel matrix	Corning	Cat# 356237
2-mercaptoethanol	ThermoFisher	Cat# 21985023
polyethylenimine	Cosmobio	Cat# 24765-100
PGI KFA001 FlexAble CoraLite(R) 488 Antibody Labeling Kit for Rabbit IgG,Trial Size 50RXN	Proteintech	Cat# KFA001
PGI KFA002 Proteintech FlexAble CoraLite Plus 555 Antibody Labeling Kitfor Rabbit IgG 50RXN	Proteintech	Cat# KFA002
PGI KFA003 FlexAble CoraLite (R) Plus 647 Antibody Labeling Kit for Rabbit IgG, Trial Size 50RXN	Proteintech	Cat# KFA003

**Critical commercial assays**		

QIAprep Spin Miniprep Kit	Qiagen	Cat# 27106
NucleoBond® Xtra Midi Plus	TaKaRa Bio	Cat# 740412
QIAquick PCR Purification Kit	Qiagen	Cat# 28104
QIAamp DNA Mini Kit	QIAGEN	Cat# 51304
RNeasy Mini Kit	QIAGEN	Cat# 74104
DNA-free™ DNA Removal Kit	ThermoFisher	Cat# AM1906
ChIP-IT® Express	Active Motif	Cat# 53008
Lactate Assay Kit-WST	Fujifilm Wako	Cat# 349-09283
Glucose Assay Kit-WST	Fujifilm Wako	Cat# 346-09411
Bullet Blocking One for Western Blotting	nacalai tesque	Cat# 13779-01
Can Get Signal® Immunoreaction Enhancer Solution 1&2	TOYOBO	Cat# NKB-101
Clarity Western ECL Substrate	BIORAD	Cat# 1705060
BulkLysis (Erythrocyte lysing solution)(fixative-free)	Funakoshi	Cat# 60-00050-13
MEM Non-Essential Amino Acids Solution (100X)	ThermoFisher	Cat# 11140050
Lymphoprep™	VERIATS STEMCELL	Cat# 07851
RosetteSep Human T cell Enrichment Cocktail	VERIATS STEMCELL	Cat# ST-15061
TRIzol™ Reagent	ThermoFisher	Cat# 15596026
SuperScript® Reverse Transcriptase	ThermoFisher	Cat# 18080093
TB Green Premix Ex Taq II (Tli RNase H Plus)	TaKaRa Bio	Cat# RR82WR
Cellstain Hoechst 33342 Solution (1 mg/ml H2O)	Fujifilm Wako	Cat# 346-07951
Millicell®-24 Cell Culture Insert Plate	Sigma-Aldrich	Cat# PSET010
Pierce™ BCA Protein Assay Reagent B	ThermoFisher	Cat# 23224
0.45 μm-membrane filters	Merck Millipore	Cat# HAWP04700

**Deposited data**		

RNA sequencing data of surgically resected liver cancer	Pinyol et al. (2021)	TCGA: PanCancer Atlas
RNA sequencing data of surgically resected HCC	Shimada et al. (2019)	TMDU dataset
RNA sequencing data of Acvr2a NC and KO mouse HCC cells	This paper	GEO: GSE248922
RNA sequencing data of Acvr2a NC and KO mouse HCC cells	This paper	Science DB: https://doi.org/10.57760/sciencedb.21374
scRNA-seq data	Gene Expression Omnibus site	GEO: GSE125449, GEO: GSE146115, GEO: GSE149614, GEO: GSE151530, GEO: GSE189903, GEO: GSE242889

**Experimental models: Cell lines**		

Human: HepG2	ATCC	Cat# HB-8065™
Human: HLF	JCRB Cell Bank	Cat# JCRB0405
Human: HLE	JCRB Cell Bank	Cat# JCRB0404
Human: JHH4	JCRB Cell Bank	Cat# CVCL_2787
Human: JHH5	JCRB Cell Bank	Cat# CVCL_0364
Human: HuH7	JCRB Cell Bank	Cat# CVCL_0336
Human: PLC/PRF/5	ATCC	Cat# CRL-8024
Human: HEK293T	ATCC	Cat# CRL-1573
Mouse: Hepa1-6	ATCC	Cat# CRL-1830
Mouse: 3H3	Lab preserve	N/A

**Experimental models: Organisms/strains**		

Mouse: KSN/Slc	SANKYO LABO	N/A
Mouse: C57BL/6	SANKYO LABO	N/A

**Recombinant DNA**		

lentiGuide-Puro vector	Addgene	Cat# 52963
lentiCRISPR v2 vector	Addgene	Cat# 52961
pCMVΔR8.2	Addgene	Cat# 12263
pCMV-VSV-G	Addgene	Cat# 8454
pLV[shRNA]-Hygro-U6>hLDHA [shRNA#1]	VectorBuilder	Cat# VB900132-3087sgz
pLV[shRNA]-Hygro-U6>hLDHA [shRNA#2]	VectorBuilder	Cat# VB900132-3088hvp
pLV[shRNA]-Hygro-U6>mLdha [shRNA#1]	VectorBuilder	Cat# VB900132-3089gvs
pLV[shRNA]-Hygro-U6>mLdha [shRNA#2]	VectorBuilder	Cat# VB900132-3090mdx
pLV[Exp]-Puro-CMV>tTS/rtTA	VectorBuilder	Cat# VB010000-4479gwh
pLV[miR30]-Hygro-TRE>ORF_Stuffer: {hSLC16A3[miR30-shRNA#1]}	VectorBuilder	Cat# VB220908-1329kuk
pLV[miR30]-Hygro-TRE>ORF_Stuffer: {hSLC16A3[miR30-shRNA#2]}	VectorBuilder	Cat# VB220908-1331umr
pLV[miR30]-Hygro-TRE>ORF_Stuffer: {mSLC16A3[miR30-shRNA#1]}	VectorBuilder	Cat# VB220908-1333zzw
pLV[miR30]-Hygro-TRE>ORF_Stuffer: {mSLC16A3[miR30-shRNA#2]}	VectorBuilder	Cat# VB220908-1336mkz

**Oligonucleotides**		

Primers for qPCR	This paper	see Table S3

**Software and algorithms**		

Fiji/ImageJ		https://imagej.net/Fiji
R	CRAN	https://www.r-project.org/
Python		https://www.python.org/
GraphPad Prism 8	GraphPad Prism	https://www.graphpad.com/scientific-software/prism/
GSEA 4.1.0	Broad Institute	N/A
WinMDI 2.8		WinMDI 2.8 Download (Free) - Winmdi.exe (informer.com)

**Other**		

Illumina NovaSeq 6000 instrument	Rhelxia	N/A
StepOne real-time PCR system	ThermoFisher	N/A
FACSCalibur	BD Biosciences	N/A
IN Cell Analyzer 2000	GE Healthcare	N/A
LAS-4000 mini	Fujifilm Wako	N/A
centrifugal filtration device	Pall	OD010C33

### Experimental model and study participant details

#### Patients and samples

A total of 194 patients underwent curative resection for HCC at Tokyo Medical and Dental University Hospital between 2013 and 2019, and 44 of them received FGD-PET/CT. The SUVmax-high ad -low groups were determined based on the median. The study included adults aged 37 to 93 years, comprising 152 males and 42 females. Health status, prior treatment, and other clinical information were written in Table S3. All the patients provided informed consent before enrollment and were anonymously coded in accordance with ethical guidelines. All patients provided written informed consent before sampling, according to the Declaration of Helsinki. This study was performed in a blinded manner and was approved by Tokyo Medical and Dental University Hospital Ethics Committee (permission number G2017-018).

#### Animal models

All animal experiments were conducted in accordance with ethical guidelines and were approved by the Tokyo Medical and Dental University Hospital Animal Experimental Ethics Committee (permission number A2023-003C3). The study utilized male C57BL/6 and KSN nude mice, aged 4 to 5 weeks. Mice were housed in a controlled environment, with a maximum of four animals per cage, monitored daily to ensure cleanliness and sufficient food and water. The temperature was maintained at 22 ± 2°C, with a relative humidity of 50–60%. A 12-h light/dark cycle was implemented (lights on from 08:00 to 20:00), with appropriate light intensity. Noise levels were minimized, and ammonia concentrations were regularly monitored to ensure a clean and stress-free environment.

#### Cell lines

HEK293T, HepG2, HLF, HLE, JHH4, JHH5, HuH7, PLC/PRF/5, and Hepa1-6 cells were purchased from American Type Culture Collection (ATCC). Mouse cell line 3H3 was derived from a liver tumor grown in a C57BL/6 MC4R-KO mouse fed with high-fat diet and harbored *Hras*^Q61L^ mutation.^50^ Cells were cultured in DMEM medium (Wako, Osaka, Japan) supplemented with 10% fetal bovine serum (FBS) and maintained in a humidified incubator at 37°C with 5% CO2 and harvested using 0.05% trypsin-0.03% EDTA (Wako). PBMCs were collected from healthy volunteers, and isolated by density gradient centrifugation with Lymphoprep (VERITAS STEMCELL Technologies, Vancouver, CA) and RosetteSep Human T cell Enrichment Cocktail (VERITAS STEMCELL Technologies). Human T cells were cultured in AIM-V (Thermo fisher Scientific, Waltham, MA) containing 10% heat-inactivated human AB serum (Gemini Bio-Products, West Sacramento, CA). Murine T cells were harvested from the spleen of C57BL/6 mice, and cultured in RPMI1640 (Wako) containing 10% heat-inactivated FBS, 100 U/ml penicillin, 100 mg/mL streptomycin, 0.05 mM 2-mercaptoethanol, 0.1 mM MEM nonessential amino acids, 1 mM sodium pyruvate, and 10 mM L-HEPES (all from Thermo Fischer Scientific). Cells were checked for mycoplasma contamination using TaKaRa PCR Mycoplasma Detection Set (TaKaRa Bio, Shiga, Japan).

### Method details

#### Tumor seeding and explant culture

After suspended in 100 μL Matrigel (BD Biosciences, San Jose, CA), 1×10^5^ to 1×10^6^ cells were subcutaneously inoculated into KSN nude mice and C57BL/6 mice. Tumor-bearing mice were sacrificed, and the tumor tissues were immediately minced into aliquots under sterile conditions. After decolonized at 4°C overnight in culture medium, the small pieces were explanted on culture dishes. An orthotopic tumor transplantation model was based on our previous report.^51^ Briefly, after suspended in 20 μL Matrigel, 5×10^6^ cells were directly injected into the mouse liver through a small incision under anesthesia, and then the incision was closed with a 6-0 silk suture.

#### *In vivo* treatments

Seven days after subcutaneous inoculation of 1×10^5^ cells into mice, anti-PD-1, anti-CTLA4, anti-CD25 or IgG isotype control antibody (200 μg/head; Bio X Cell) was intraperitoneally injected into the tumor-bearing mice every seven days. Following the similar protocol, the MCT4 inhibitor VB124 (30 mg/kg; MedChemExpress, Monmouth Junction, NJ) was orally administered twice a day.

#### Bioinformatics analysis

Transcriptome and clinical data of a total of 370 HCC patients, comprising 153 viral cases and 217 non-viral cases, were obtained from the TCGA through the cBioPortal website. The rates of gene mutations were analyzed independently for viral and non-viral cases, and the difference between the two groups was evaluated using χ2 test. The overall survival of the viral and non-viral HCC groups was compared using the log rank test to determine the maximum difference in the P-values (ΔlogP = logPviral - logPnon-viral).

#### Single-cell analysis

Raw count data of six scRNA-seq datasets (GSE125449, GSE146115, GSE149614, GSE151530, GSE189903 and GSE242889) were downloaded from the Gene Expression Omnibus site. The gene expression data of HCC samples were read, integer-transformed, filtered with min_genes = 200 and min_cells = 5, normalized with target_sum = 1e4 and then log-transformed in Scanpy. Cell types were predicted using the CellTypist program with the “Healthy_Human_Liver.pkl” and “Immune_ALL_Low.pkl” models. For quality control, cells were excluded if any of the following metrics exceeded five mean absolute deviations: log1p_n_genes_by_counts (log-transformed number of genes expressed in the count matrix), log1p_total_counts (log-transformed total counts per cell), or pct_counts_mt (percentage of mitochondrial gene counts). In all cells, after 3,000 highly variable genes were selected using “seurat_v3” algorithm and scaled, PCA and Harmony integration were conducted. For “cells labeled as “Hepatocytes” by the CellTypist program, after 3,000 highly variable genes were selected using “seurat_v3” algorithm and scaled, PCA and Harmony integration were conducted. Single-sample gene set enrichment analysis (ssGSEA) of scRNA-seq data was performed with the MSigDB gene sets using the scGSVA package in R. HCC samples were divided into glycolysis-high and -low groups based on the median enrichment score for the glycolytic pathway, and the ratio of cytotoxic T cell (Tcm/Naive cytotoxic T cells, Tem/Trm cytotoxic T cells, Tem/Temra cytotoxic T cells and Trm cytotoxic T cells) to Treg cell (Regulatory T cells) was estimated in each sample.

#### Genome engineering

CRISPR target sequences for gene knockout are provided in the key resources table. The oligos were cloned into either the lentiGuide-Puro vector (#52963; Addgene, Watertown, MA) or the lentiCRISPR-Bsd vector, which was derived from the lentiCRISPR v2 vector (#52961; Addgene), following the provided instructions. Lentiviral vectors for expressing shRNA against *Ldha*, *Mct4,* and *Mct1* were purchased from VectorBuider (Chicago). HEK293T cells were transfected with the lentiviral transfer plasmids, pCMVΔR8.2 and pCMV-VSV-G, using polyethylenimine (Polysciences, Warrington, PA). After two to three days of transfection, culture supernatants were collected and filtered through 0.45 μm-membrane filters (Merck Millipore, Burlington, MA). The cells were infected with the collected supernatant for 12 h and then treated with antibiotics such as 10 μg/mL puromycin (Thermo Fisher Scientific), 10 μg/mL blasticidin S (Wako), or 300 μg/mL hygromycin (Thermo Fisher Scientific) for a period of two days. Cells with tetracycline-inducible shRNA expression were cultured in medium including 500 μg/mL doxycycline (Sigma-Aldrich, St. Louis, MO) and grown in the KSN and C57BL/6 mice fed with or without doxycycline in drinking water (2 mg/mL).

The entire coding sequences of *Ldha* and *Mct4* were amplified from the cDNA of Hepa1-6 cells using the primer pair sets, 5′- TCGACTCGAGGCCACCATGAGTAAGTCCTCAGGCGG-3' (forward)/5′- ATCCGCGGCCGCTTAGAACTGCAGCTCCTTCTG-3' (reverse) and 5′-TCGACTCGAGGCCACCATGGTGAAGAAGGAAAAAACG-3' (forward)/5′-ATCCGCGGCCGCTTAGGTTTTCAAGGGCTTCATG-3' (reverse), respectively. The PCR products were digested with *Xho*I and *Not*I, and cloned into the CSII-EF-MCS-IRES-Hygro lentiviral plasmid.

#### RNA-seq analysis

Sequencing libraries were generated from total RNA utilizing the TruSeq Standard mRNA Library Kit (Illumina, San Diego, CA). Subsequently, RNA sequencing was conducted on the NovaSeq 6000 system (Ilumina) by Rhelixa (Tokyo, Japan). The sequence reads were aligned to the mouse reference genome (GRCm38) using STAR (version 2.7.0days), and quantified using RSEM (version 1.3.1). Differentially expressed genes were identified using DESeq2 (version 1.14.1). Enriched pathways were identified using the Gene Set Enrichment Analysis (GSEA). Volcano plots were generated using EnhancedVolcano (version 1.20.0). To predict the biological functions and signaling pathways of DEGs, we used the Database for Annotation, Visualization, and Integrated Discovery (DAVID) tool to analyze their enrichment in Gene Ontology (GO) terms and Kyoto Encyclopedia for Genes and Genomes (KEGG) pathways.

#### Knockdown experiments

Two siRNAs and negative control (Mission siRNA Universal Negative Control) were purchased from Merck KGaA (Frankfurter Strasse, Germany). Each siRNA was transfected into HCC cells to give a final concentration of 50 nM using the Lipofectamine RNAiMAX Transfection Reagent (Invitrogen, Carlsbad, CA) according to the manufacturer’s instructions. After 48 to 72 h of culture, transfected cells were harvested and used for gene expression and functional analysis.

#### DNA extraction, PCR analysis, and sanger sequencing analysis

Cell pellets were resuspended in TNE Buffer (consisting of 10 mM Tris–HCl, pH 8.0; 150 mM NaCl; 2 mM EDTA; 0.5% SDS) supplemented with 1% proteinase K (TaKaRa Bio), and the suspension was incubated at 55°C overnight. Genomic DNA was subsequently extracted using the phenol-chloroform extraction method. The primer sets and conditions for PCR amplification are shown in the key resources table. Following PCR amplification, the products were purified using the QIAquick PCR Purification Kit (QIAGEN, Hulsterweg, Netherlands), and subjected to direct sequencing by Azenta (Burlington, MA).

#### RNA extraction and real-time reverse transcription PCR (qRT-PCR)

Total RNA was extracted from cellular specimens using the TRIzol Reagent (Thermo Fisher Scientific), and the removal of any contaminating DNA was achieved through the digestion process utilizing the DNA-free DNA Removal Kit (Thermo Fisher Scientific). To synthesize single-stranded complementary DNA, 1 μg of total RNA was reverse-transcribed employing the SuperScript III Reverse Transcriptase (Thermo Fisher Scientific). Quantitative RT-PCR analysis was conducted using the TB Green Premix Ex Taq II (TaKaRa Bio) in conjunction with the StepOne real-time PCR system (Thermo Fisher Scientific), following the guidelines provided by the manufacturer. The ΔΔCt method was employed for relative quantification, and 18S ribosome RNA was used as an internal control. The primer sets for PCR are shown in the Table S5.

#### Flow cytometric analysis

1×10^5^ cells of human and mouse lymphocytes were co-cultured with 1×10^4^ cells of tumor cells at 37°C for 12 h and carefully washed with 2% FBS-PBS. Two days after exposure to VB124, AR-C155858 and lactate, cells were collected. Tumor tissues were thoroughly minced using a scalpel, and then incubated at 37°C for 1 h in a mixture of DNase I (Wako) and trypsin. After neutralization with medium containing FBS, cells were filtered using Falcon Cell Strainer 35 μm (Corning, NY). These cells were stained using antibodies shown in the key resources table. Intracellular Foxp-3 staining was conducted utilizing Foxp-3/Transcription Factor Staining Buffer Set (Thermo Fisher Scientific) in accordance with the manufacturer’s protocol. Intracellular GZMB staining was also performed following the similar protocol. The fluorescence intensity was assessed using FACSCalibur (BD Biosciences) and FACSlyric (BD Biosciences), and the percentage was calculated employing WinMDI 2.8 and FACSuite Clinical Software (BD Biosciences). All experiments were conducted in triplicate.

#### Proliferation assay

Cell viability was evaluated using the Cell Counting Kit-8 (Dojindo, Kumamoto, Japan). Briefly, cells were plated at a density of 1.0×10^3^ cells per well in 96-well plates. Cells were incubated in a fresh culture medium containing 10% Cell Counting Kit-8 reagent for 2 h under 5% CO2 at 37°C. Absorbance was evaluated at 450 nm using a spectrophotometer (iMark; BioRad Laboratories, Hercules, CA).

#### Colony formation assay

Cells were seeded at a density of 1 × 10^3^ cells per well in 6 well plates and incubated at 37°C. After a duration of 10–14 days, the cells were fixed using 100% methanol and counterstained for nuclei using crystal violet solution (Wako). The quantification of stained cells was accomplished utilizing ImageJ 1.54 software and Fiji (Windows 64-bit).

#### Cell migration and invasion assays

The double-chamber migration and invasion assays were conducted using transwell chambers (24-well plate, 8-μm pores; BD Biosciences). Following the addition of 0.8 mL of culture medium to the lower chambers, cells were seeded onto the upper chambers at a density of 3–5 × 10^4^ cells per well in 0.3 mL of serum-free medium. The cells were then incubated at 37°C for 24 to 48 h. After the cells on the upper surface of the filters were removed using cotton wool swabs, the remaining cells on the lower surface were fixed with 100% methanol, stained with Giemsa solution, and quantified by examining ten randomly selected high-magnification fields (×100) for each sample.

#### Sphere formation assay

Cells were seeded at a density of 1 × 10^3^ cells per well in 6-well plates and incubated at 37°C. Cultured medium included 20 ng/mL of EGF (epithelial growth factor), 20 ng/mL of bFGF (fibroblast growth factor ), and B27 supplement (Proteintech, Rosemont, IL). After a duration of 10–14 days, the number of spheres was counted by examining ten randomly selected high-magnification fields (×100) for each sample.

#### Chromatin immunoprecipitation analysis

Chromatin immunoprecipitation analysis was conducted utilizing the ChIP-IT Express Kit (Active Motif, Carlsbad, CA) following the manufacturer’s protocol. As a negative control, normal rabbit IgG (#2729; Cell Signaling Technology) was employed. The primer sets and amplification conditions for quantitative PCR targeting the promoter region and gene body of human and mouse *LDHA* are specified in the key resources table. The enrichment of immunoprecipitated DNA was normalized to the input.

#### Western blotting

Upon collecting whole cell lysates using ice-cold RIPA buffer (Thermo Fisher Scientific), 10 to 20 μg of protein from each sample was subjected to electrophoresis on 10% sodium dodecyl sulfate-polyacrylamide gels and subsequently transferred onto Immobilon polyvinyldifluoride membranes (Merck Millipore). The membrane was then blocked with Bullet Blocking One for Western Blotting (Nacalai tesque, Kyoto, Japan) at room temperature for 5 min, and incubated for an hour at 37°C with primary antibodies and Can Get Signal Solution 1 (TOYOBO, Osaka, Japan). Next, secondary antibodies were applied with Can Get Signal Solution 2 (TOYOBO) at room temperature for an hour, and signals were detected using Clarity Western ECL Substrate (Bio-Rad, Hercules, CA) with LAS-4000 mini (Fujifilm, Tokyo, Japan). β-actin served as an internal control. The antibodies used for Western blot analysis are summarized in the key resources table.

#### Co-immunoprecipitation (Co-IP)

The Universal Magnetic Co-IP Kit (Active Motif, Carlsbad, CA) was used according to the manufacturer’s protocol. Briefly, after cell debris were removed by centrifugation at 12,000 g for 20 min at 4°C, 5 μg of cell lysate was mixed with 2 μg of anti-Hif1α antibody and gently rotated overnight at 4°C. Protein G magnetic beads were added to the mixture and incubated for 2 h at 4°C to capture the immune complexes. Beads were then washed 3 to 5 times with ice-cold wash buffer, and the immune complexes were eluted by boiling the beads in SDS sample buffer for 5 min at 95°C. The subsequent steps followed the same procedure as described for Western blot analysis.

#### Immunohistochemical analysis

All tumor samples were collected from surgical resection in our institution. The tissues were fixed overnight in Mildform 20N (Wako), subsequently embedded in paraffin, and sectioned at a thickness of 4 μm. The sections were then immersed in sodium citrate buffer (pH 6.0) for antigen retrieval, and incubated overnight at 4°C with primary antibodies as shown in the key resources table. The sections were probed with peroxidase-labeled anti-mouse or anti-rabbit IgG antibody (Histofine Simple Stain MAX-PO, Nichirei Bioscience, Tokyo, Japan) and visualized using diaminobenzidine (Wako). Finally, the nuclei were stained with hematoxylin. The staining intensity was classified as 0 (absence of staining), 1+ (low intensity), 2+ (moderate intensity), or 3+ (high intensity) by two researchers independently. Tumor samples with a score of 0 or 1 and with a score of 2 or 3 were categorized into the low and high expression groups, respectively. CD8-positive and Foxp-3-positive cells were counted in three high-magnification microscopic fields (100x), and the mean values were calculated.

#### Immunofluorescent analysis

Multiplex immunofluorescence staining was performed using FlexAble CoraLite Plus 488, 555 and 647 Antibody Labeling Kits for Rabbit IgG (Proteintech) according to the manufacturer’s protocols. Briefly, rabbit IgG antibodies were conjugated with the fluorophores CoraLite 488, 555 and 647. The sections were immersed in sodium citrate buffer (pH 6.0) for antigen retrieval, and incubated for 4 h at 4°C with the fluorescent-labeled antibodies. The stained sections were subsequently counterstained and mounted using ProLong Gold Antifade Mountant (Thermo Fisher Scientific). The slides were viewed with a fluorescent microscope (Carl Zeiss, Oberkochen, Germany).

#### Measurement of lactate and glucose concentration

Extracellular lactate concentration was assessed utilizing the Lactate Assay Kit-WST (Funakoshi, Tokyo, Japan) following the manufacturer’s designated protocol. Forty eight hours after a total of 5 × 10^4^ cells were plated onto a 6-cm dish, the medium was collected to measure lactate concentration. Intracellular lactate concentration was determined using a centrifugal filtration device (OD010C33; Pall, Tokyo, Japan). Cells were prepared at a density of 1 x 10^6^, and subsequent measurements were conducted in accordance with the manufacturer’s protocol. Similarly, extracellular and intracellular glucose concentration was assessed utilizing the Glucose Assay Kit-WST (Funakoshi).

### Quantifications and statistical analysis

The methods for statistical testing were described in each figure legends. Data are presented as means of triplicates and SD unless otherwise indicated. For multivariate analysis, variables found to be significant in univariate analyses (*p* < 0.05) and/or considered important on the basis of logical and/or biomedical grounds were entered into the logistic regression model to identify factors independently associated with survival by the forced entry, as appropriate. Furthermore, covariates were evaluated by stepwise forward and backward selection methods with a cut-off P-value of 0.10. GraphPad Prism8 (GraphPad Software, San Diego, California, USA) and R version 4.3.1 (R Foundation for Statistical Computing, Vienna, Austria) were used for statistical analyses and all graph plotting. Significance was concluded at ∗*p* < 0.05, ∗∗*p* < 0.01, ∗∗∗*p* < 0.001.

## Supplemental information

Document S1. Figures S1–S18 and Tables S1–S5

Document S2. Article plus supplemental information
