# Supplementary material for: ACVR2A attenuation impacts lactate production and hyperglycolytic conditions attracting regulatory T cells in hepatocellular carcinoma
Source: Cell Rep Med. 2025 Mar 25;6(4):102038. doi: 10.1016/j.xcrm.2025.102038 (PMC12047472; doi:10.1016/j.xcrm.2025.102038)
Supplement: Document S1. Figures S1–S18 and Tables S1–S5 [file mmc1.pdf]

Cell Reports Medicine, Volume *6*

Supplemental information

ACVR2A attenuation impacts lactate production and hyperglycolytic conditions attracting regulatory T cells in hepatocellular carcinoma

Koya Yasukawa, Shu Shimada, Yoshimitsu Akiyama, Tomohiko Taniai, Yosuke Igarashi, Shu Tsukihara, Yoshiaki Tanji, Kentaro Umemura, Atsushi Kamachi, Atsushi Nara, Masahiro Yamane, Keiichi Akahoshi, Akira Shimizu, Yuji Soejima, Minoru Tanabe, and Shinji Tanaka

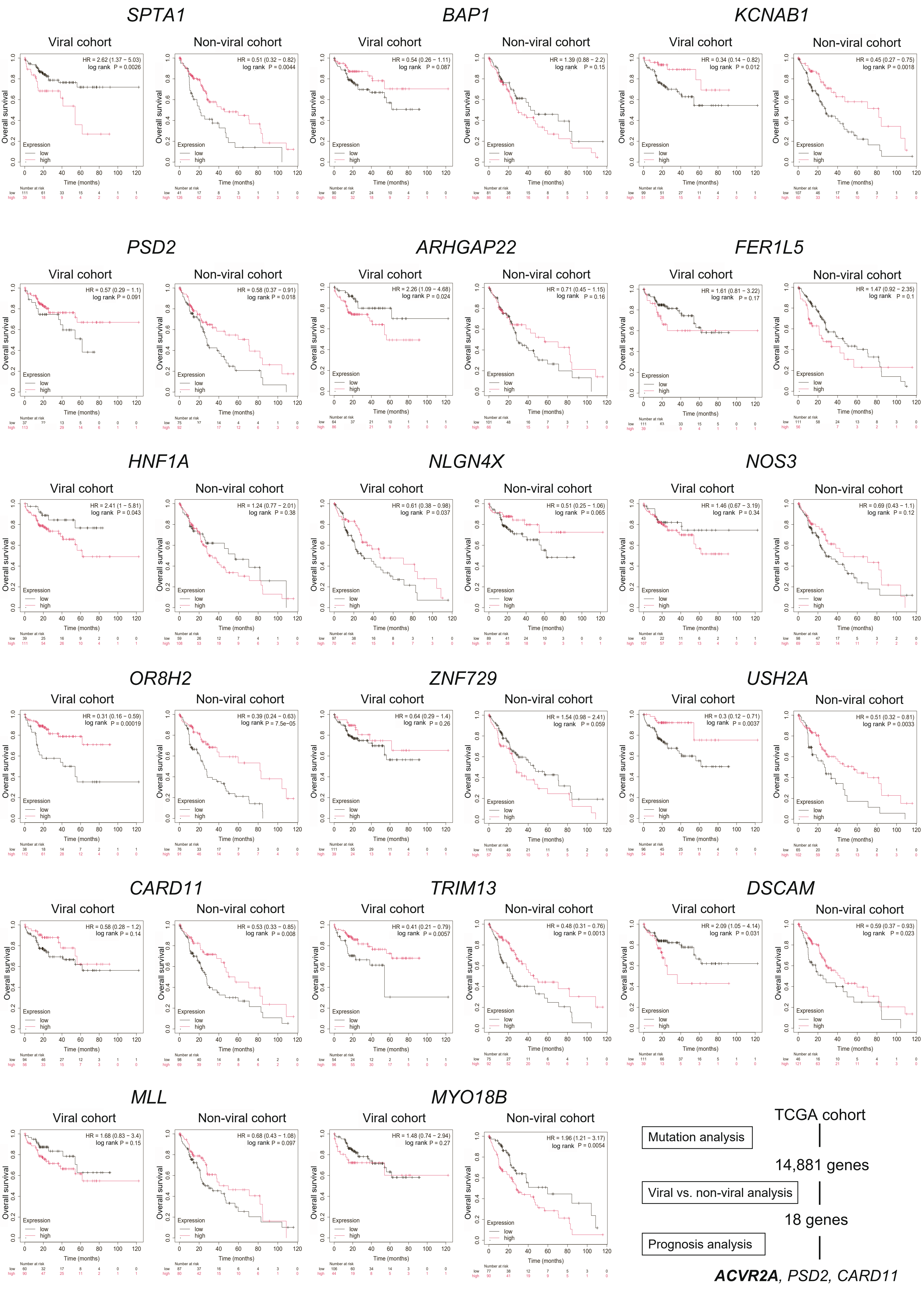

SPTA1
Viral cohort
HR = 2.62 (1.37 − 5.03) log rank P = 0.0026
Non-viral cohort
HR = 0.51 (0.32 − 0.82) log rank P = 0.0044
BAP1
Viral cohort
HR = 0.54 (0.26 − 1.11) log rank P = 0.087
Non-viral cohort
HR = 1.39 (0.88 − 2.2) log rank P = 0.15
KCNAB1
Viral cohort
HR = 0.34 (0.14 − 0.82) log rank P = 0.012
Non-viral cohort
HR = 0.45 (0.27 − 0.75) log rank P = 0.0018
PSD2
Viral cohort
HR = 0.57 (0.29 − 1.1) log rank P = 0.091
Non-viral cohort
HR = 0.58 (0.37 − 0.91) log rank P = 0.018
ARHGAP22
Viral cohort
HR = 2.26 (1.09 − 4.68) log rank P = 0.024
Non-viral cohort
HR = 0.71 (0.45 − 1.15) log rank P = 0.16
FER1L5
Viral cohort
HR = 1.61 (0.81 − 3.22) log rank P = 0.17
Non-viral cohort
HR = 1.47 (0.92 − 2.35) log rank P = 0.1
HNF1A
Viral cohort
HR = 2.41 (1 − 5.81) log rank P = 0.043
Non-viral cohort
HR = 1.24 (0.77 − 2.01) log rank P = 0.38
NLGN4X
Viral cohort
HR = 0.61 (0.38 − 0.98) log rank P = 0.037
Non-viral cohort
HR = 0.51 (0.25 − 1.06) log rank P = 0.065
NOS3
Viral cohort
HR = 1.46 (0.67 − 3.19) log rank P = 0.34
Non-viral cohort
HR = 0.69 (0.43 − 1.1) log rank P = 0.12
OR8H2
Viral cohort
HR = 0.31 (0.16 − 0.59) log rank P = 0.00019
Non-viral cohort
HR = 0.39 (0.24 − 0.63) log rank P = 7.5e−05
ZNF729
Viral cohort
HR = 0.64 (0.29 − 1.4) log rank P = 0.26
Non-viral cohort
HR = 1.54 (0.98 − 2.41) log rank P = 0.059
USH2A
Viral cohort
HR = 0.3 (0.12 − 0.71) log rank P = 0.0037
Non-viral cohort
HR = 0.51 (0.32 − 0.81) log rank P = 0.0033
CARD11
Viral cohort
HR = 0.58 (0.28 − 1.2) log rank P = 0.14
Non-viral cohort
HR = 0.53 (0.33 − 0.85) log rank P = 0.008
TRIM13
Viral cohort
HR = 0.41 (0.21 − 0.79) log rank P = 0.0057
Non-viral cohort
HR = 0.48 (0.31 − 0.76) log rank P = 0.0013
DSCAM
Viral cohort
HR = 2.09 (1.05 − 4.14) log rank P = 0.031
Non-viral cohort
HR = 0.59 (0.37 − 0.93) log rank P = 0.023
MLL
Viral cohort
HR = 1.68 (0.83 − 3.4) log rank P = 0.15
Non-viral cohort
HR = 0.68 (0.43 − 1.08) log rank P = 0.097
MYO18B
Viral cohort
HR = 1.48 (0.74 − 2.94) log rank P = 0.27
Non-viral cohort
HR = 1.96 (1.21 − 3.17) log rank P = 0.0054
Overall survival
Time (months)
Expression
low
high
Number at risk
TCGA cohort
Mutation analysis
14,881 genes
Viral vs. non-viral analysis
18 genes
Prognosis analysis
ACVR2A, PSD2, CARD11

Supplementary Figure 1. -----related to Figure 1.

Kaplan-Meier curves of OS in the high and low expression groups in each gene. The schematic representation of the screening analysis for exploring genes specifically mutated and significantly associated with unfavorable outcomes in non-viral HCC was shown in the bottom right panel. The P-value was calculated by the log-rank test.

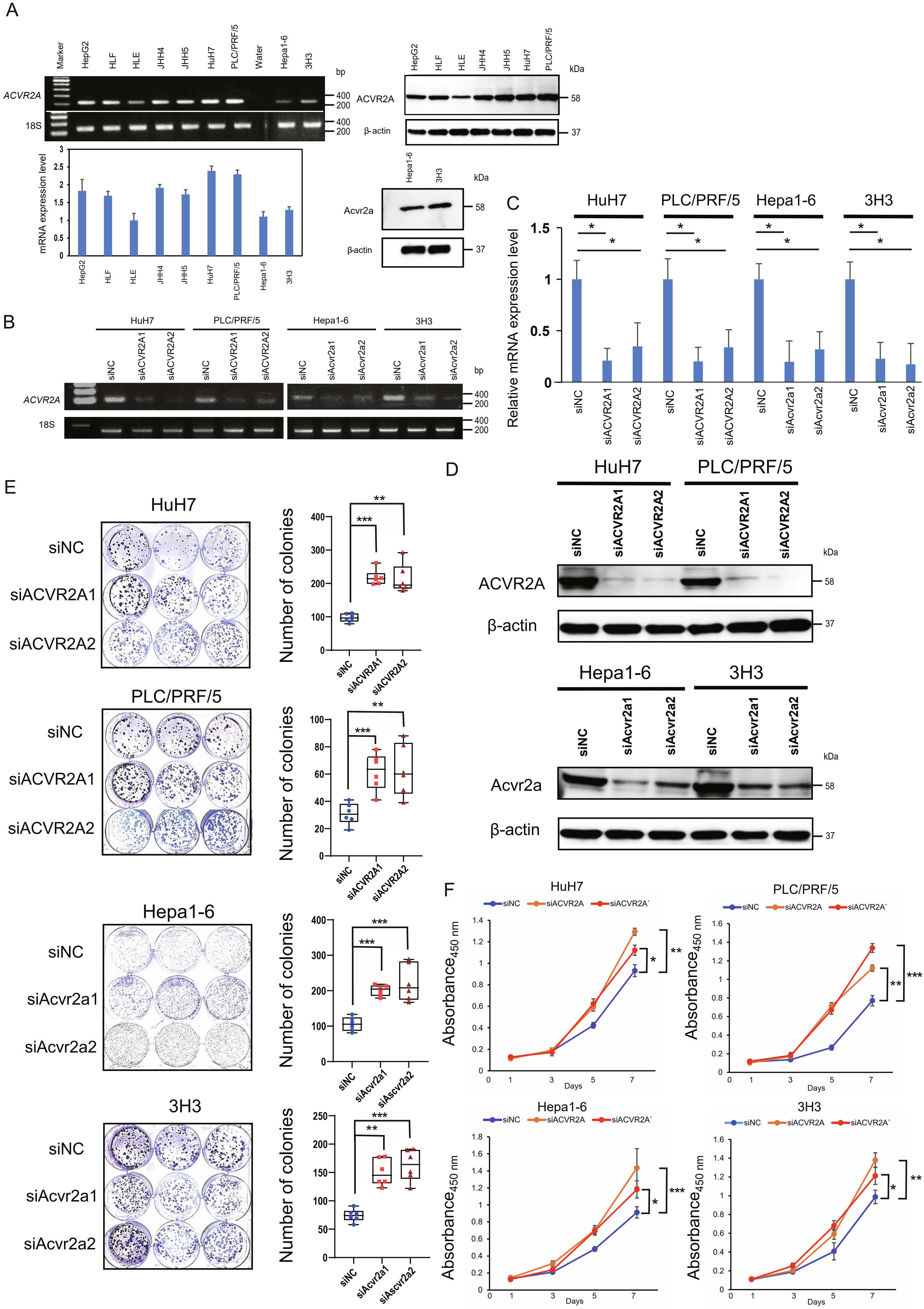

A
Marker
HepG2
HLF
HLE
JHH4
JHH5
HuH7
PLC/PRF/5
Water
Hepa1-6
3H3
bp
ACVR2A
18S
400
200
mRNA expression level
ACVR2A
β-actin
kDa
58
37
Acvr2a
B
HuH7
PLC/PRF/5
Hepa1-6
3H3
siNC
siACVR2A1
siACVR2A2
siAcvr2a1
siAcvr2a2
C
Relative mRNA expression level
D
E
Number of colonies
siAscvr2a2
F
siACVR2A
siACVR2A`
Absorbance450 nm
Days

Supplementary Figure 2. -----related to Figure1.

(A) Semi-quantitative and quantitative RT-PCR analysis and Western blot analysis of ACVR2A expression levels in human and mouse HCC cell lines. 18S ribosome RNA and β-actin were used as internal controls for RT-PCR and Western blot analysis, respectively.

(B, C, D) Semi-quantitative (B) and quantitative (C) RT-PCR analysis and Western blot analysis (D) of *ACVR2A* expression levels in HCC cells with *ACVR2A* knockdown. Bars represent relative mRNA levels compared to cells transfected with siNC. The P-value was calculated by ANOVA with Tukey-Kramer post hoc test. 18S ribosome RNA and β-actin were used as internal controls for RT-PCR and Western blot analysis, respectively.

(E, F) Colony formation (E) and proliferation (F) assays of HuH7, PLC/PRF/5, Hepa1-6, and 3H3 cells. Representative photo images in each assay were included. The P-value was calculated using Kruskal-Wallis test with Steel-Dwass post hoc test (E). The P-value was calculated by Welch's *t* test (F).

Boxes represent the 25th, 50th, and 75th percentiles. Data are the mean ± SD. *$p < 0.05$, **$p < 0.01$, ***$p < 0.001$.

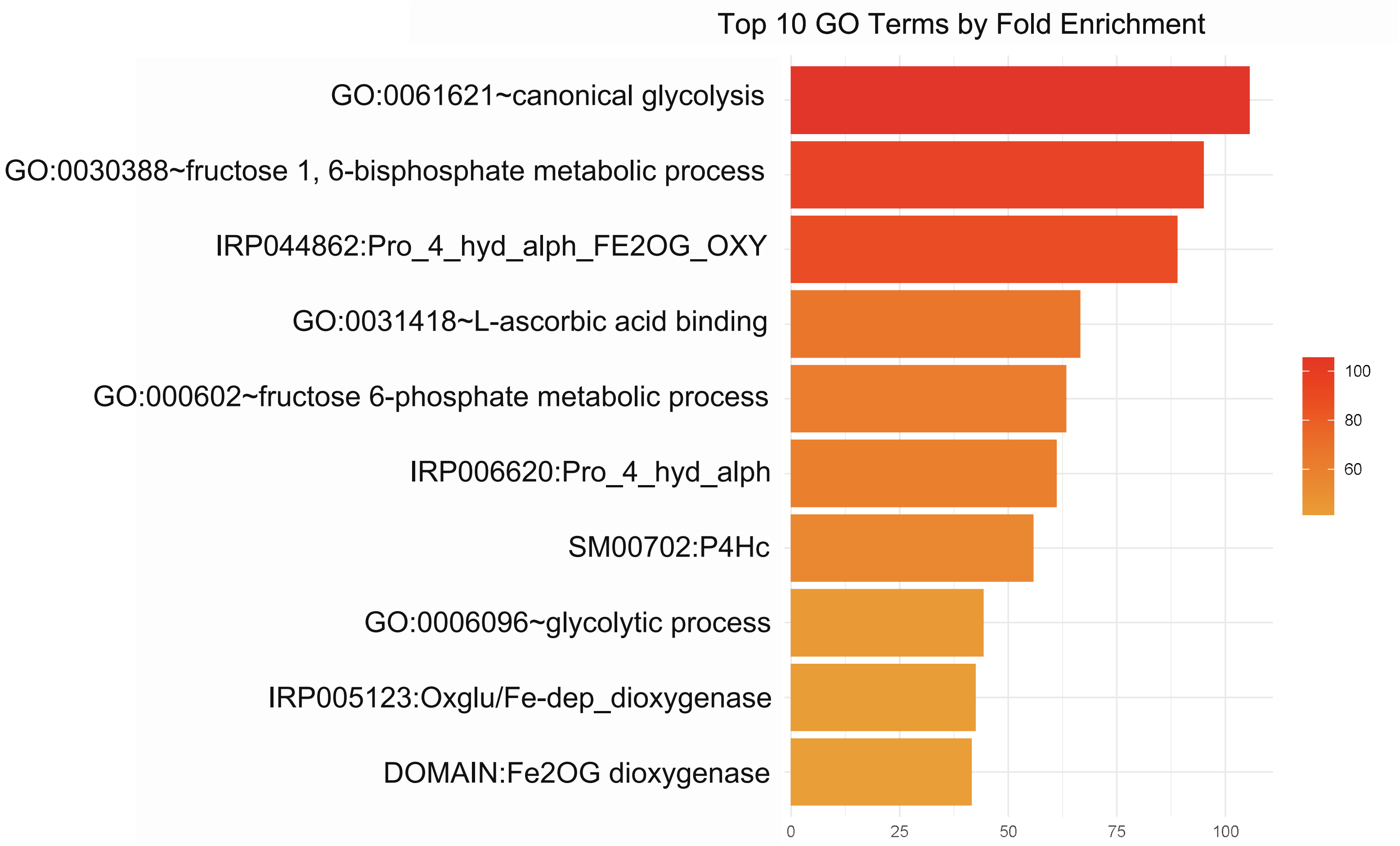

Top 10 GO Terms by Fold Enrichment
GO:0061621~canonical glycolysis
GO:0030388~fructose 1, 6-bisphosphate metabolic process
IRP044862:Pro_4_hyd_alph_FE2OG_OXY
GO:0031418~L-ascorbic acid binding
GO:000602~fructose 6-phosphate metabolic process
IRP006620:Pro_4_hyd_alph
SM00702:P4Hc
GO:0006096~glycolytic process
IRP005123:Oxglu/Fe-dep_dioxygenase
DOMAIN:Fe2OG dioxygenase
0
25
50
75
100
100
80
60

Supplementary Figure 3. -----related to Figure2.

Gene ontology enrichment analysis on differentially expressed genes between the Hepa1-6 NC and KO cells. The top 10 GO terms are ranked in ascending order of statistical significance calculated using the DAVID tool.

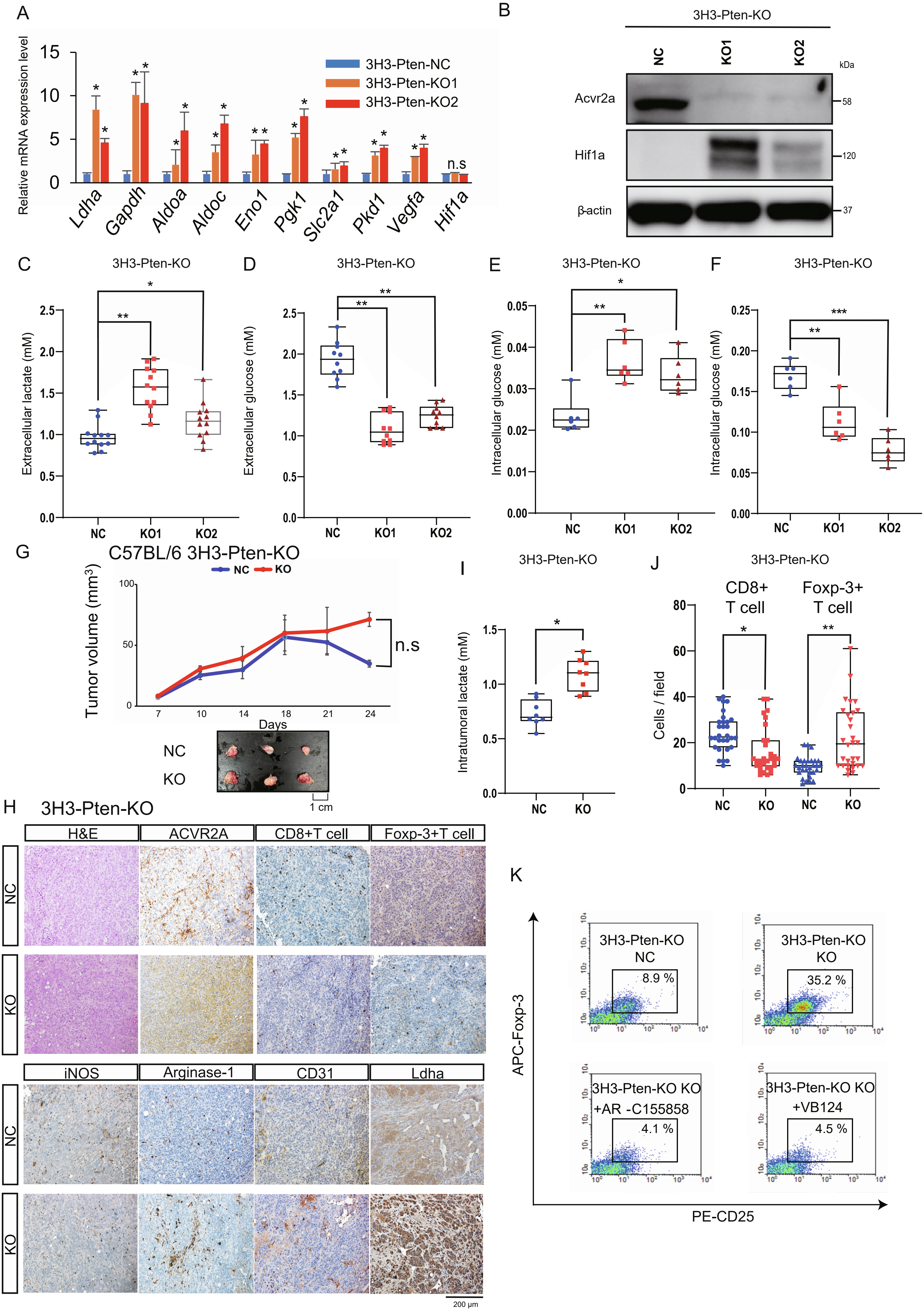

A
Relative mRNA expression level
3H3-Pten-NC
3H3-Pten-KO1
3H3-Pten-KO2
Ldha Gapdh Aldoa Aldoc Eno1 Pgk1 Slc2a1 Pkd1 Vegfa Hif1a
n.s
B
3H3-Pten-KO
NC KO1 KO2
kDa
Acvr2a
Hif1a
β-actin
58
120
37
C
3H3-Pten-KO
Extracellular lactate (mM)
NC KO1 KO2
D
3H3-Pten-KO
Extracellular glucose (mM)
NC KO1 KO2
E
3H3-Pten-KO
Intracellular glucose (mM)
NC KO1 KO2
F
3H3-Pten-KO
Intracellular glucose (mM)
NC KO1 KO2
G
C57BL/6 3H3-Pten-KO
NC KO
Tumor volume (mm³)
Days
n.s
NC
KO
1 cm
I
3H3-Pten-KO
Intratumoral lactate (mM)
NC KO
J
3H3-Pten-KO
CD8+ T cell
Foxp-3+ T cell
Cells / field
NC KO NC KO
H
3H3-Pten-KO
H&E
ACVR2A
CD8+T cell
Foxp-3+T cell
NC
KO
iNOS
Arginase-1
CD31
Ldha
200 μm
K
3H3-Pten-KO NC
8.9 %
3H3-Pten-KO KO
35.2 %
3H3-Pten-KO KO +AR -C155858
4.1 %
3H3-Pten-KO KO +VB124
4.5 %
APC-Foxp-3
PE-CD25

Supplementary Figure 4. -----related to Figure 2-6.

(A) Quantitative RT-PCR analysis of genes upregulated in 3H3-Pten-KO cells with Acvr2a knockout.

Bars represent relative mRNA levels compared to the NC cells. The P-value was calculated by ANOVA with Tukey-Kramer post hoc test.

(B) Western blot analysis of Hif1a expression levels. β-Actin was used as an internal control.

(C, D) Extracellular lactate (C) and glucose (D) levels. The P-value was calculated using Kruskal-Wallis test with Steel-Dwass post hoc test.

(E, F) Intracellular lactate (E) and glucose (F) levels. The P-value was calculated by Kruskal-Wallis test with Steel-Dwass post hoc test.

(G) Tumorigenicity assay in immunoproficient mice (N = 3). Representative photo images of tumor specimens were included. The P-value was calculated using Welch's t test.

(H) Representative immunohistochemical images of ACVR2A, LDHA and immune cell markers in tumor tissues. Nuclei were stained using hematoxylin. The scale bar represents 200 μm.

(I) Intratumoral lactate levels. The P-value was calculated using Mann-Whitney U test.

(J) Quantitative immunohistochemical analysis of CD8+ T cell and Foxp-3+ T cell infiltration. The Pvalue was calculated using Mann-Whitney U test.

(K) Flow cytometric analysis of splenocytes co-cultured with 3H3-Pten-KO cells with Acvr2a knockout.

H&E: hematoxylin and eosin. Boxes represent the 25th, 50th, and 75th percentiles. Data are the mean ±SD. n.s, not significant; $*p < 0.05$, $**p < 0.01$, $***p < 0.001$.

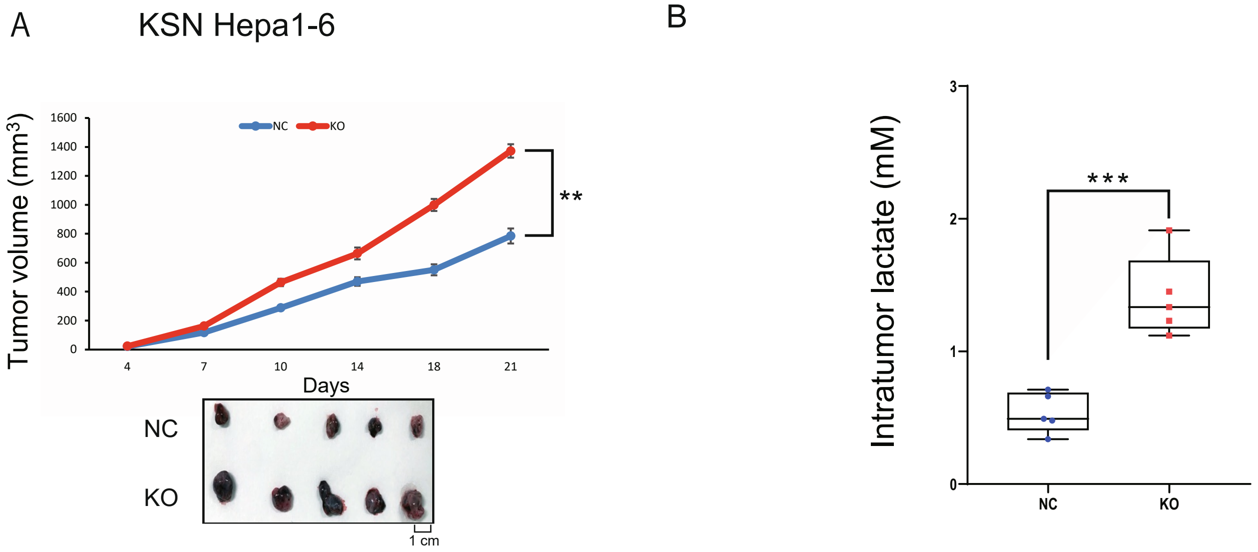
A
KSN Hepa1-6
NC
KO
1600
1400
1200
1000
800
600
400
200
0
Tumor volume (mm3)
**
4
7
10
14
18
21
Days
NC
KO
1 cm
B
3
2
1
0
Intratumor lactate (mM)
***
NC
KO

Supplementary Figure 5. -----related to Figure 3.

(A) Tumorigenicity assay of Hepa1-6 KO cells in immunodeficient mice (N = 6). Representative photo images of tumor specimens were included. The P-value was calculated by Welch's *t* test.

(B) Intratumoral lactate levels. The P-value was calculated using Mann-Whitney *U* test.

Boxes represent the 25th, 50th, and 75th percentiles. Data are the mean ± SD. n.s, not significant; $^{**}p < 0.01$, $^{***}p < 0.001$.

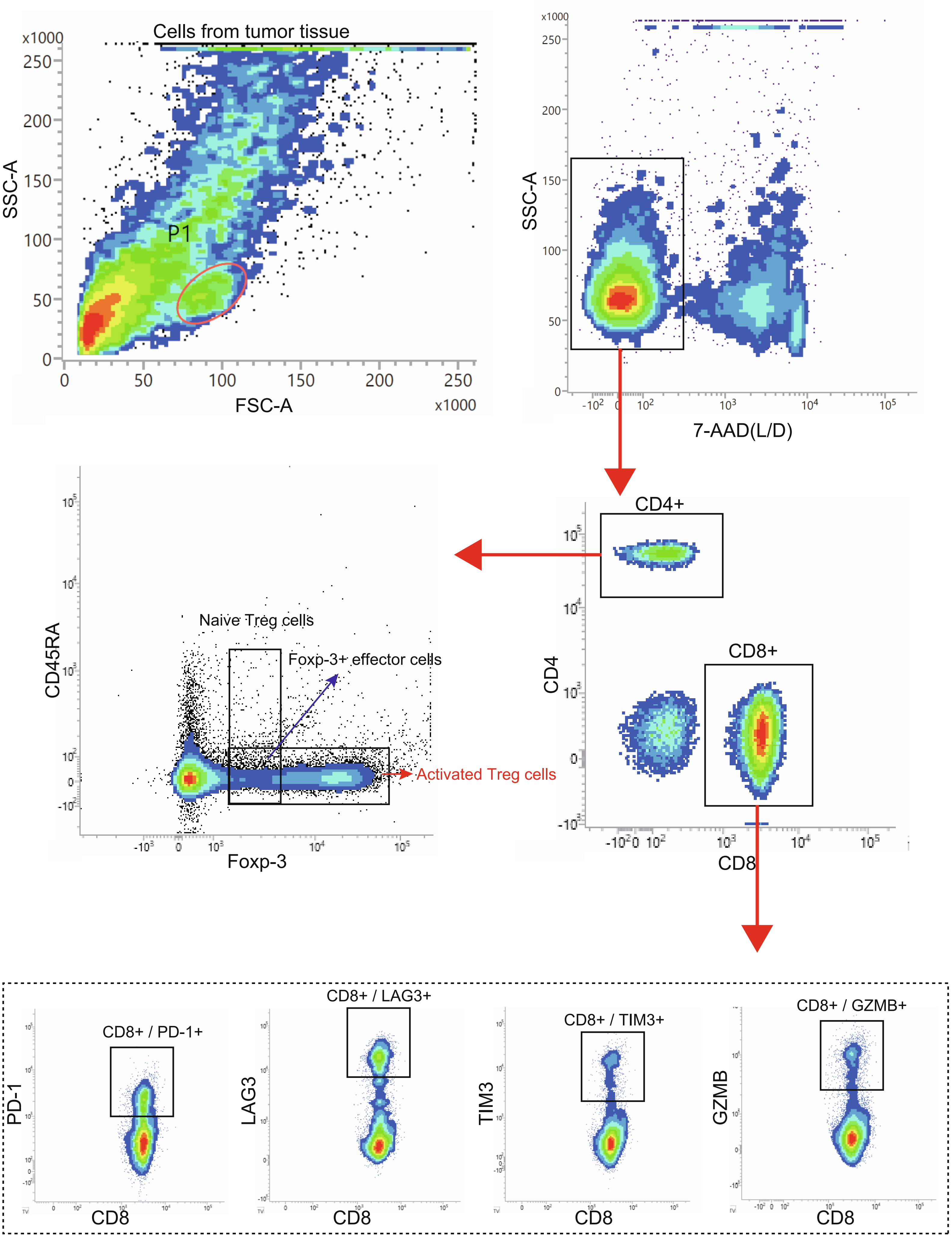

Cells from tumor tissue
SSC-A
FSC-A
P1
7-AAD(L/D)
CD4+
CD8+
CD4
CD8
Naive Treg cells
Foxp-3+ effector cells
Activated Treg cells
CD45RA
Foxp-3
CD8+ / PD-1+
CD8+ / LAG3+
CD8+ / TIM3+
CD8+ / GZMB+
PD-1
LAG3
TIM3
GZMB

Supplementary Figure 6. -----related to Figure 3-7.

Representative images of flow cytometric analysis of cells collected from culture dishes and tumor tissues.

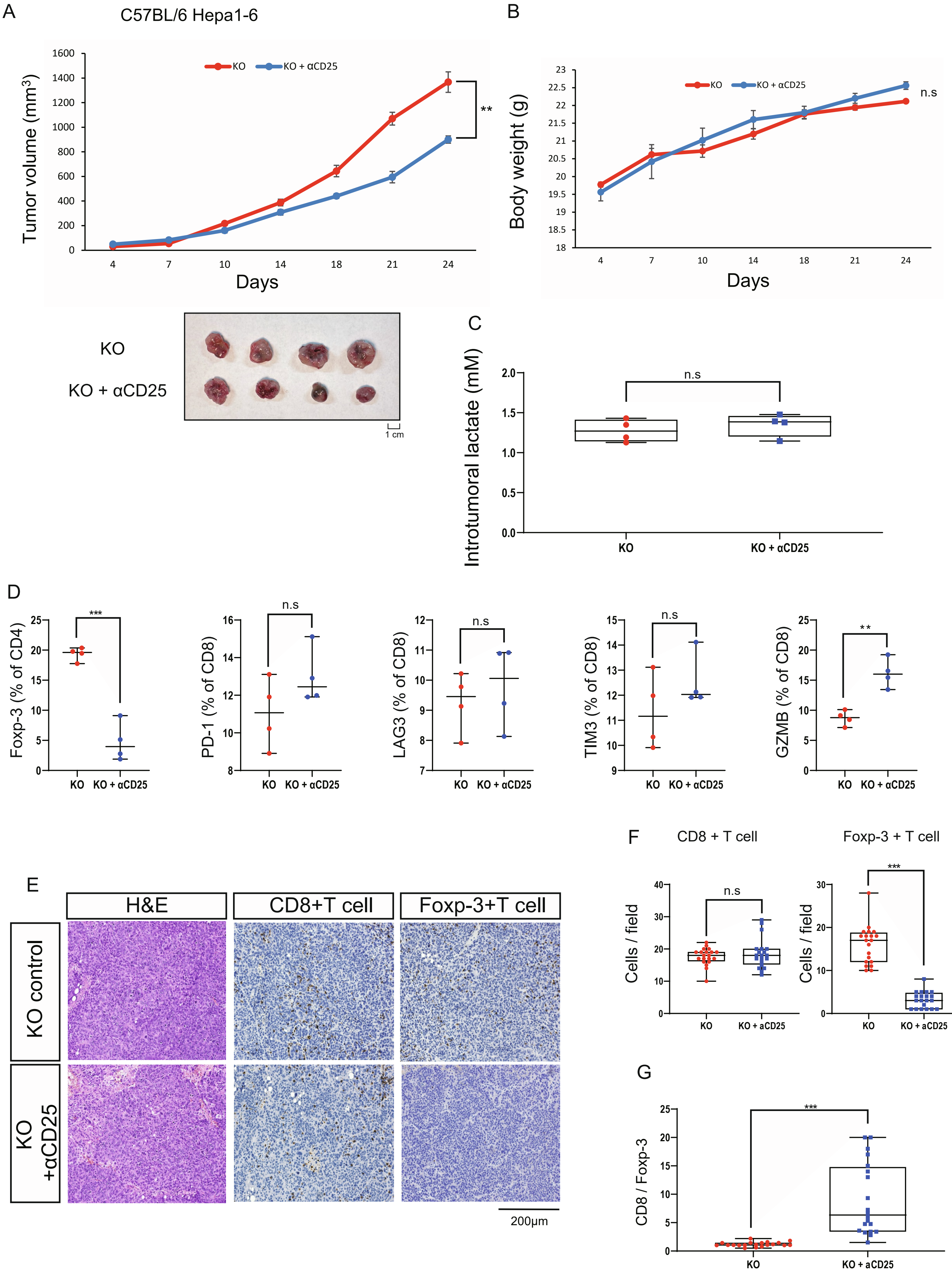

A
C57BL/6 Hepa1-6
Tumor volume (mm³)
KO
KO + αCD25
**
Days
B
Body weight (g)
KO
KO + αCD25
n.s
Days
KO
KO + αCD25
1 cm
C
Introtumoral lactate (mM)
n.s
KO
KO + αCD25
D
Foxp-3 (% of CD4)
***
PD-1 (% of CD8)
n.s
LAG3 (% of CD8)
n.s
TIM3 (% of CD8)
n.s
GZMB (% of CD8)
**
KO
KO + αCD25
E
H&E
CD8+T cell
Foxp-3+T cell
KO control
KO +αCD25
200μm
F
CD8 + T cell
Foxp-3 + T cell
Cells / field
n.s
***
KO
KO + aCD25
G
CD8 / Foxp-3
***
KO
KO + aCD25

Supplementary Figure 7. -----related to Figure 3.

(A) Tumorigenicity assay of Hepa1-6 KO cells in immunoproficient mice treated with anti-CD25 antibody (N = 4). Representative photo images of tumor specimens were included. The P-value was calculated by Welch's t test.

(B) Body weight of mice treated with anti-CD25 antibody. The P-value was calculated using Kruskal-Wallis test with Steel-Dwass post hoc test.

(C) Intratumoral lactate levels in tumors derived from Hepa1-6 cells treated with anti-CD25 antibody. The P-value was calculated using Mann-Whitney U test.

(D) Quantitative flow cytometric analysis of Foxp-3+ Treg cells and CD8+ T cells with exhaustion and activation markers. The P-value was calculated using Mann-Whitney U test.

(E) Representative immunohistochemical images of CD8+ T cells and Foxp-3+ T cells. Nuclei were stained with hematoxylin. H&E: hematoxylin and eosin. The scale bar represents 200 μm.

(F) Quantitative immunohistochemical analysis of CD8+ T cell and Foxp-3+ T cell infiltration. The P-value was calculated using Mann-Whitney U test.

(G) CD8+ T cell / Foxp-3+ Treg cell ratio. The P-value was calculated using Mann-Whitney U test. Boxes represent the 25th, 50th, and 75th percentiles. Data are the mean ± SD. n.s, not significant; $*p < 0.05$, $**p < 0.01$, $***p < 0.001$.

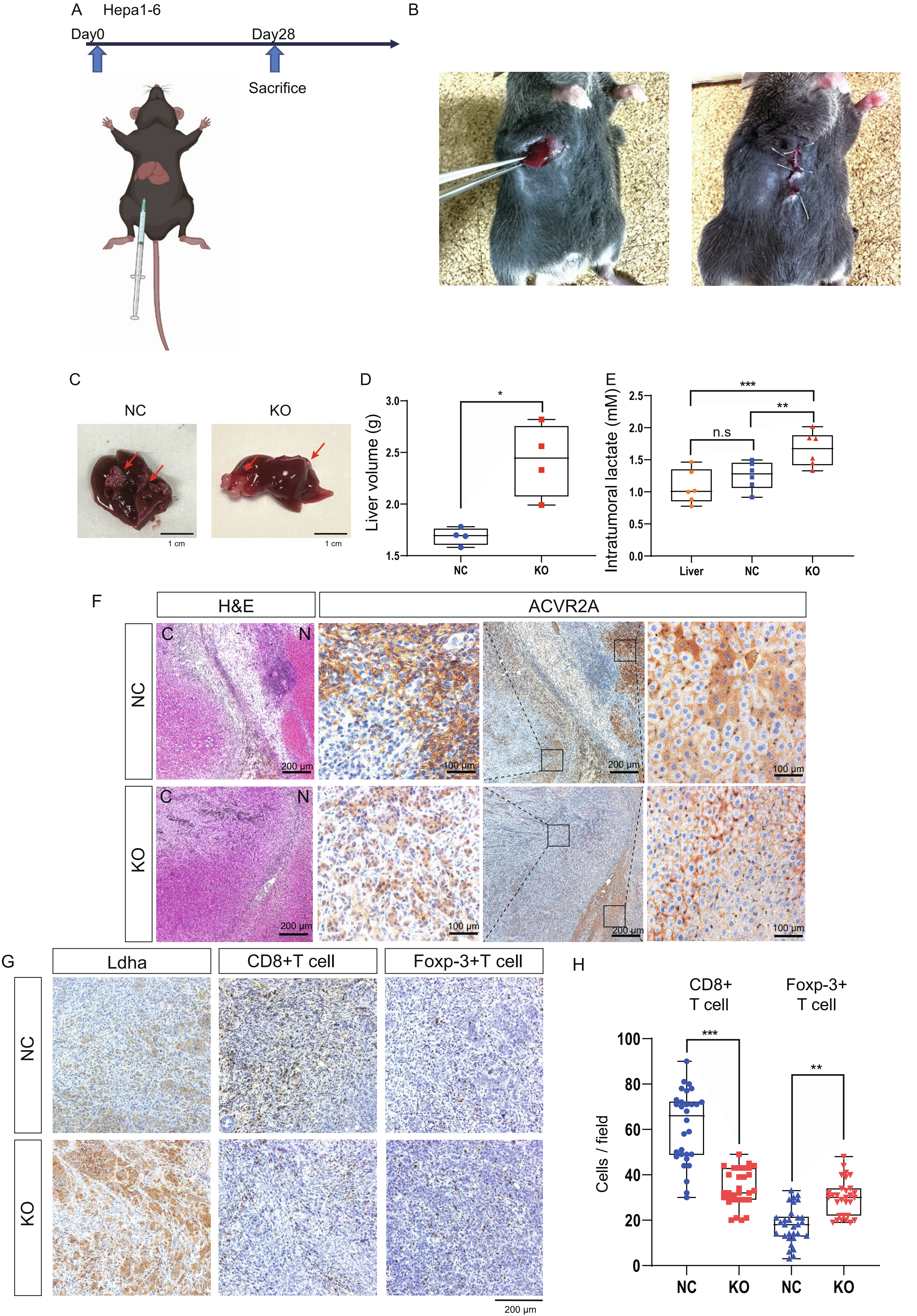
A
Hepa1-6
Day0
Day28
Sacrifice
B
C
NC
KO
1 cm
1 cm
D
Liver volume (g)
*
3.0
2.5
2.0
1.5
NC
KO
E
Intratumoral lactate (mM)
***
**
n.s
2.5
2.0
1.5
1.0
0.5
0.0
Liver
NC
KO
F
H&E
ACVR2A
NC
KO
C
N
200 μm
100 μm
G
Ldha
CD8+T cell
Foxp-3+T cell
NC
KO
200 μm
H
CD8+
T cell
Foxp-3+
T cell
***
**
Cells / field
100
80
60
40
20
0
NC
KO
NC
KO

Supplementary Figure 8. -----related to Figure 5.

(A, B) Schematic representation (A) and photo images (B) of orthotopic transplantation.

(C) Representative photo images of orthotopic xenografts. Red arrows indicate tumor lesions.

(D) Liver volume containing transplanted tumors. The P-value was calculated by Welch's t test.

(E) Intratumoral lactate levels in transplanted tumor tissues and normal liver tissues. The P-value was calculated using Kruskal-Wallis test with Steel-Dwass post hoc test.

(F, G) Representative immunohistochemical images of ACVR2A (F), LDHA and immune cell markers (G). Nuclei were stained using hematoxylin. C: cancerous tissues. N: adjacent liver tissues. The scale bar represents 100 or 200 μm.

(H) Quantitative immunohistochemical analysis of CD8+ T cell and Foxp-3+ T cell infiltration. The P-value was calculated using Mann-Whitney U test. H&E: hematoxylin and eosin.

Boxes represent the 25th, 50th, and 75th percentiles. Data are the mean ±SD. *p < 0.05, **p < 0.01, ***p < 0.001.

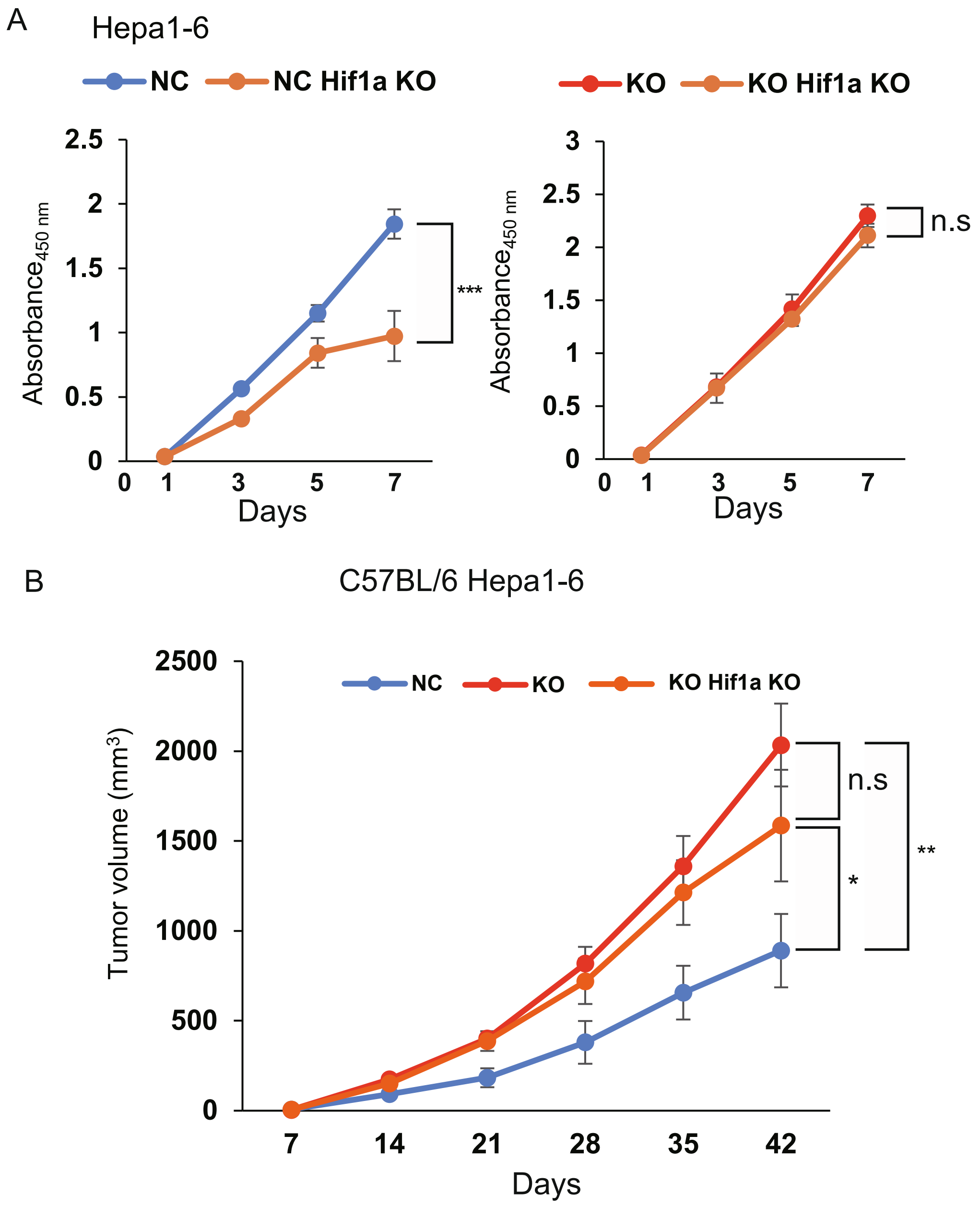
A
Hepa1-6
NC
NC Hif1a KO
KO
KO Hif1a KO
Absorbance450 nm
Days
***
n.s
B
C57BL/6 Hepa1-6
NC
KO
KO Hif1a KO
Tumor volume (mm3)
Days
n.s
*
**

Supplementary Figure 9. -----related to Figure 5.

(A) Proliferation assays of Hepa1-6 NC and KO cells with *Hif1a* knockout. The P-value was calculated by Welch’s *t* test.

(B) Tumorigenicity assay in immunoproficient mice (N = 4). The P-value was calculated using Welch's *t* test.

Data are the mean ± SD. n.s, not significant; *p < 0.05, **p < 0.01, ***p < 0.001.

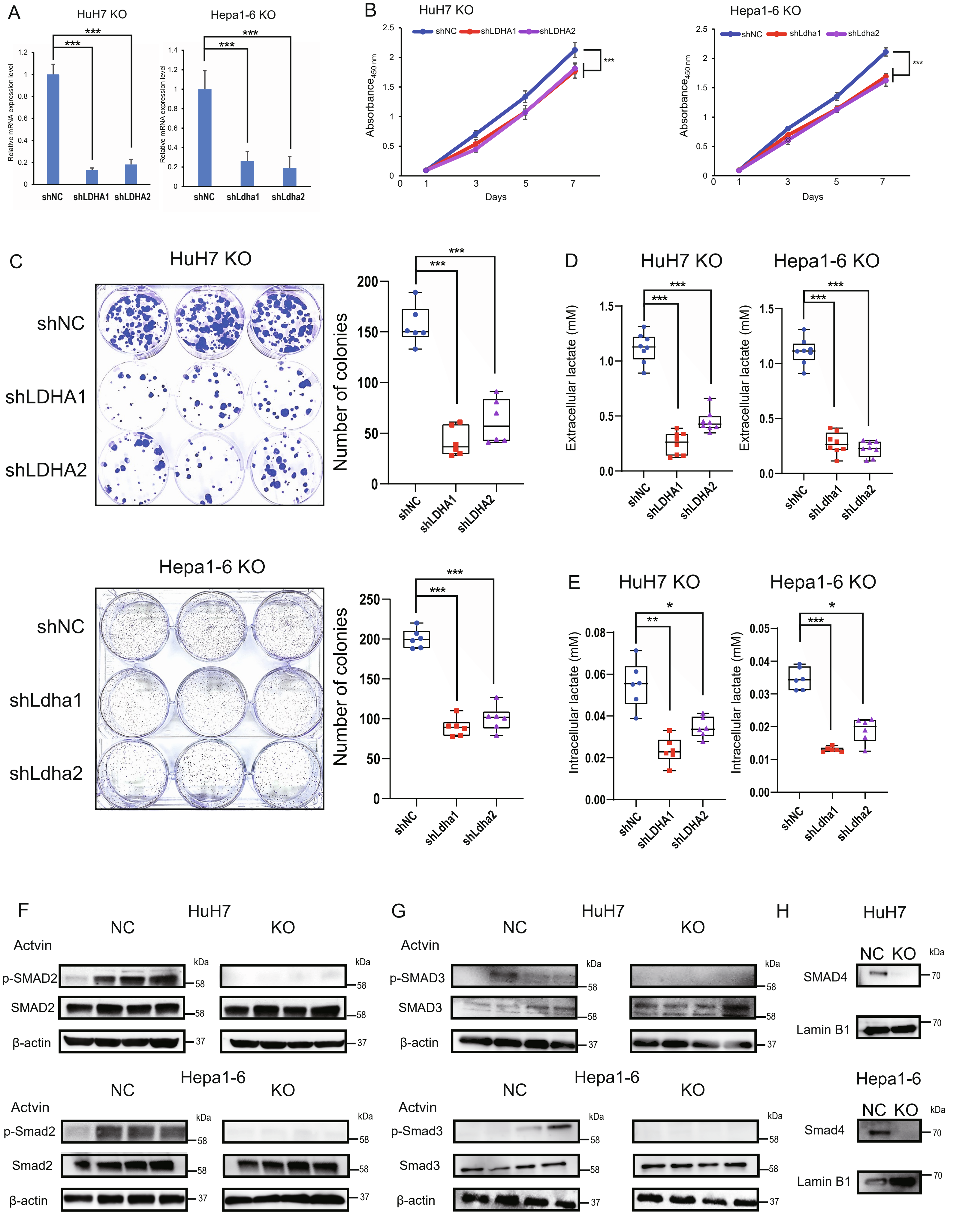

A
HuH7 KO
Hepa1-6 KO
Relative mRNA expression level
shNC
shLDHA1
shLDHA2
shLdha1
shLdha2
***
B
HuH7 KO
Hepa1-6 KO
Absorbance450 nm
Days
C
HuH7 KO
Hepa1-6 KO
Number of colonies
D
Extracellular lactate (mM)
E
Intracellular lactate (mM)
F
HuH7
Hepa1-6
NC
KO
Actvin
p-SMAD2
SMAD2
β-actin
p-Smad2
Smad2
kDa
G
p-SMAD3
SMAD3
p-Smad3
Smad3
H
SMAD4
Lamin B1
Smad4

Supplementary Figure 10. -----related to Figure 5.

(A) Quantitative RT-PCR analysis of *LDHA* expression levels in HuH7 and Hepa1-6 KO cells with *LDHA* knockdown. Bars represent relative mRNA levels compared to cells infected with shNC. The P-value was calculated by ANOVA with Tukey-Kramer post hoc test.

(B) Proliferation assays. The P-value was calculated by ANOVA with Tukey-Kramer post hoc test.

(C) Colony formation assays. Representative photo images in each colony formation assay were included. The P-value was calculated using Kruskal-Wallis test with Steel-Dwass post hoc test.

(D, E) Extracellular (D) and intracellular (E) lactate levels. The P-value was calculated using Kruskal-Wallis test with Steel-Dwass post hoc test.

(F, G) Western blot analysis of phosphorylated SMAD2 and SMAD3 under treatment with activin. β-Actin was used as an internal control.

(H) Western blot analysis of nuclear-localized SMAD4. Lamin B1 was used as an internal control for nuclear protein.

Boxes represent the 25th, 50th, and 75th percentiles. Data are the mean ± SD. *$p < 0.05$, **$p < 0.01$, ***$p < 0.001$.

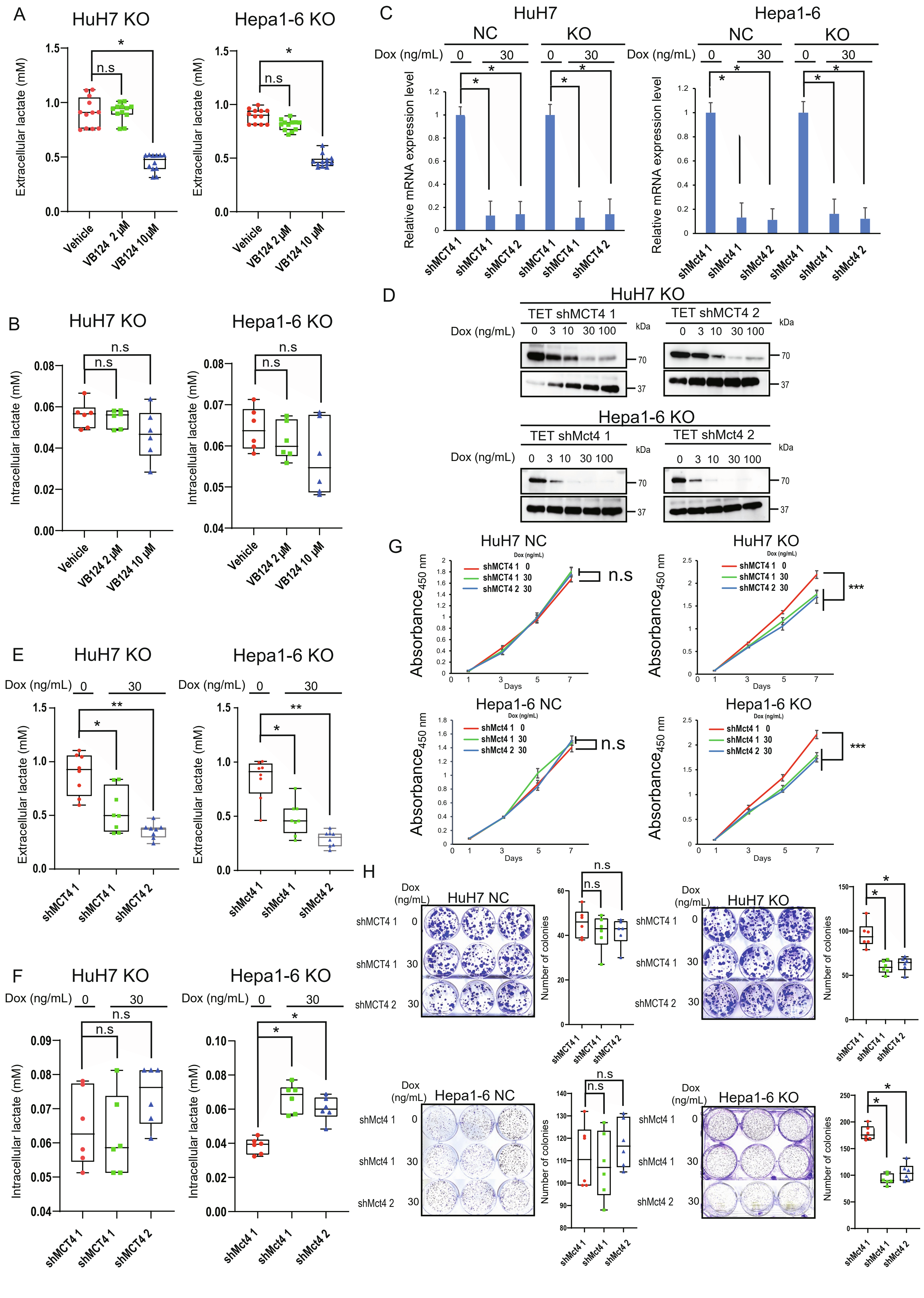
A
HuH7 KO
Hepa1-6 KO
Extracellular lactate (mM)
Vehicle
VB124 2 μM
VB124 10 μM
n.s
*
B
Intracellular lactate (mM)
C
HuH7
Hepa1-6
NC
KO
Dox (ng/mL)
0
30
Relative mRNA expression level
shMCT4 1
shMCT4 2
shMct4 1
shMct4 2
D
HuH7 KO
TET shMCT4 1
TET shMCT4 2
Dox (ng/mL)
0 3 10 30 100
kDa
70
37
Hepa1-6 KO
TET shMct4 1
TET shMct4 2
E
**
F
G
HuH7 NC
HuH7 KO
Hepa1-6 NC
Hepa1-6 KO
Absorbance450 nm
Days
Dox (ng/mL)
shMCT4 1 0
shMCT4 1 30
shMCT4 2 30
shMct4 1 0
shMct4 1 30
shMct4 2 30
***
H
Number of colonies

Supplementary Figure 11. -----related to Figure 6, 7.

(A, B) Extracellular (A) and intracellular (B) lactate levels in HuH7 and Hepa1-6 KO cells treated with VB124. The P-value was calculated using Kruskal-Wallis test with Steel-Dwass post hoc test.

(C, D) Quantitative RT-PCR (C) and Western blot (D) analyses of *MCT4* expression levels in HuH7 and Hepa1-6 cells with tetracycline-inducible *MCT4* knockdown. The P-value was calculated by ANOVA with Tukey-Kramer post hoc test. β-Actin was used as an internal control.

(E, F) Extracellular (E) and intracellular (F) lactate levels. The P-value was calculated using Kruskal-Wallis test with Steel-Dwass post hoc test.

(G) Proliferation assays. The P-value was calculated by ANOVA with Tukey-Kramer post hoc test.

(H) Colony formation assays. Representative photo images in each colony formation assay were included. The P-value was calculated using Kruskal-Wallis test with Steel-Dwass post hoc test.

Boxes represent the 25th, 50th, and 75th percentiles. Data are the mean ± SD. n.s, not significant; $^{*}p < 0.05$, $^{**}p < 0.01$, $^{***}p < 0.001$.

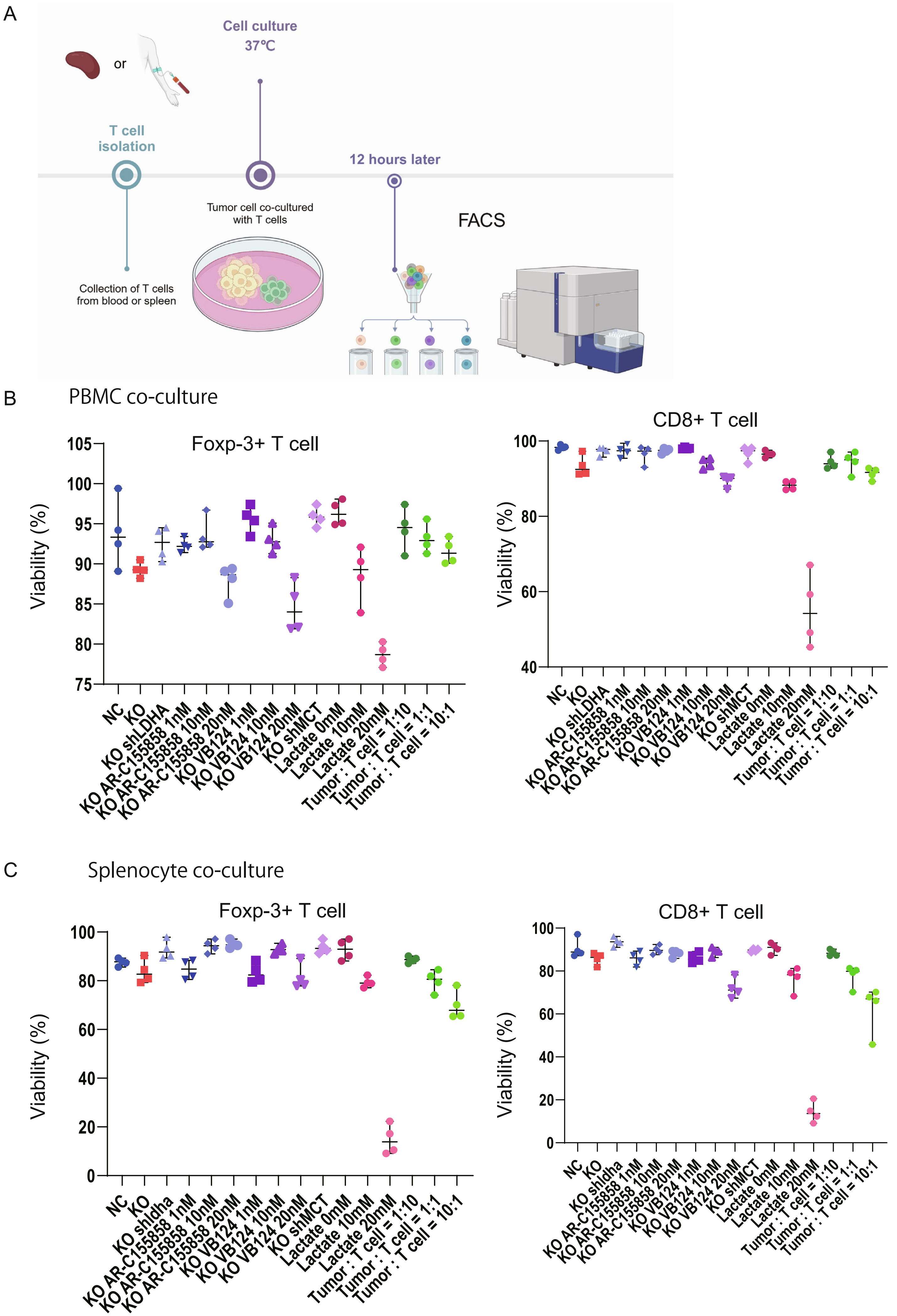
A
Cell culture
37℃
or
T cell
isolation
12 hours later
Tumor cell co-cultured
with T cells
FACS
Collection of T cells
from blood or spleen
B
PBMC co-culture
Foxp-3+ T cell
CD8+ T cell
Viability (%)
105
100
95
90
85
80
75
60
40
NC
KO
KO shLDHA
KO AR-C155858 1nM
KO AR-C155858 10nM
KO AR-C155858 20nM
KO VB124 1nM
KO VB124 10nM
KO VB124 20nM
KO shMCT
Lactate 0mM
Lactate 10mM
Lactate 20mM
Tumor : T cell = 1:10
Tumor : T cell = 1:1
Tumor : T cell = 10:1
C
Splenocyte co-culture
Foxp-3+ T cell
CD8+ T cell
Viability (%)
100
80
60
40
20
0
NC
KO
KO shldha
KO AR-C155858 1nM
KO AR-C155858 10nM
KO AR-C155858 20nM
KO VB124 1nM
KO VB124 10nM
KO VB124 20nM
KO shMCT
Lactate 0mM
Lactate 10mM
Lactate 20mM
Tumor : T cell = 1:10
Tumor : T cell = 1:1
Tumor : T cell = 10:1

Supplementary Figure 12. -----related to Figure 6.

(A) Schematic representation of the co-culture and flow cytometry of tumor cells and T lymphocytes.

(B) Quantitative flow cytometric analysis of CD8+ T cells co-cultured with HuH7 KO cells and Hepa1-6 KO cells.

(C, D) Cell viability of Foxp-3+ Treg cells and CD8+ T cells co-cultured with HuH7 KO cells (C) and Hepa1-6 KO cells (D).

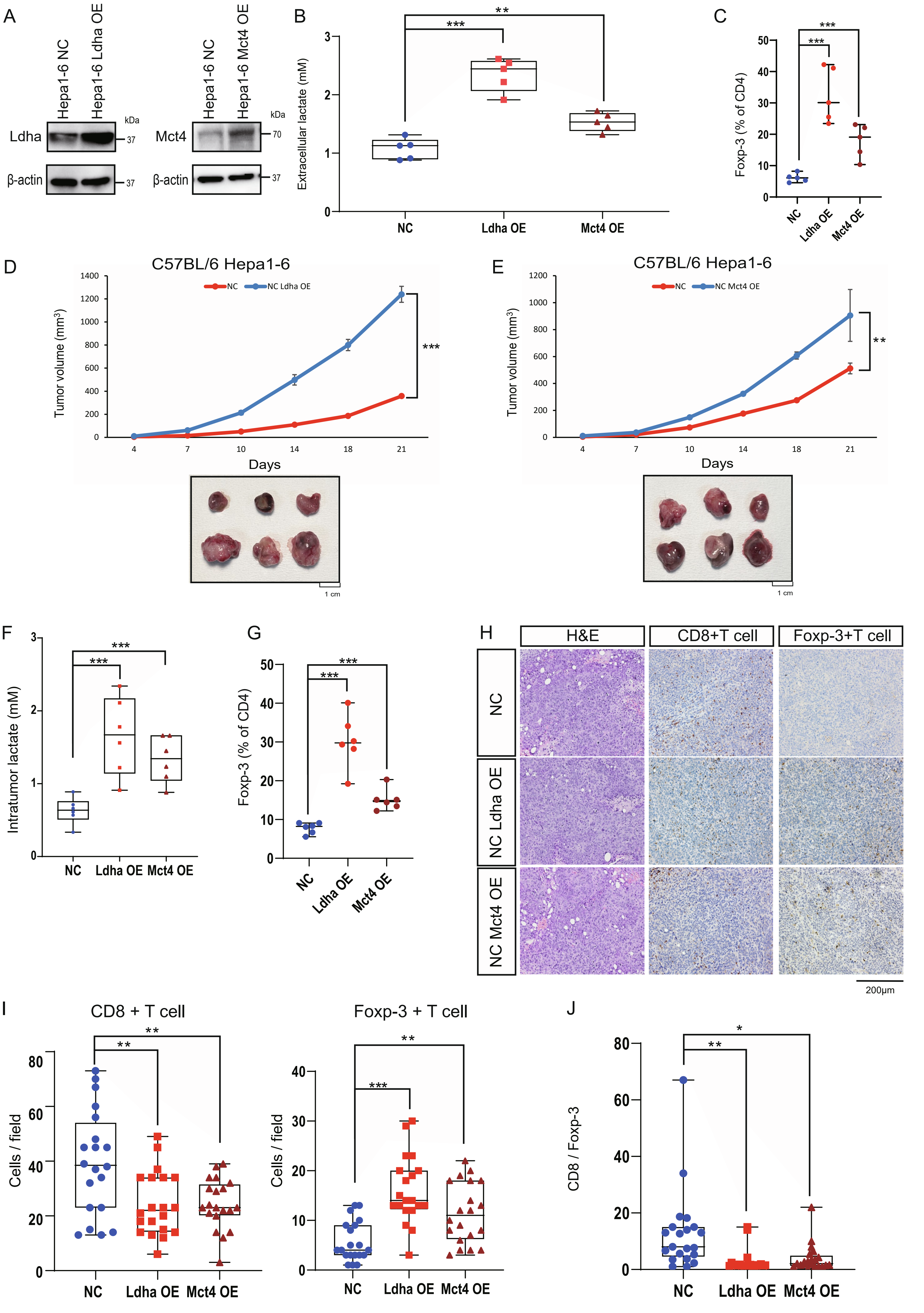
A
Hepa1-6 NC
Hepa1-6 Ldha OE
Hepa1-6 NC
Hepa1-6 Mct4 OE
kDa
Ldha
Mct4
β-actin
37
70
B
Extracellular lactate (mM)
NC
Ldha OE
Mct4 OE
C
Foxp-3 (% of CD4)
D
C57BL/6 Hepa1-6
NC
NC Ldha OE
Tumor volume (mm³)
Days
E
NC Mct4 OE
1 cm
F
Intratumor lactate (mM)
G
H
H&E
CD8+T cell
Foxp-3+T cell
NC Ldha OE
NC Mct4 OE
200μm
I
CD8 + T cell
Foxp-3 + T cell
Cells / field
J
CD8 / Foxp-3

Supplementary Figure 13. -----related to Figure 6.

(A) Western blot analysis of Hepa1-6 cells overexpressing *Ldha* and *Mct4*. β-Actin was used as an internal control.

(B) Extracellular lactate levels. The P-value was calculated by Kruskal-Wallis test with Steel-Dwass post hoc test.

(C) Quantitative flow cytometric analysis of Foxp-3+ Treg cells co-cultured with Hepa1-6 cells overexpressing *Ldha* and *Mct4*. The P-value was calculated using Kruskal-Wallis test with Steel-Dwass post hoc test.

(D, E) Tumorigenicity assay of Hepa1-6 cells overexpressing *Ldha* and *Mct4* in immunoproficient mice (N = 3). Representative photo images of tumor specimens were included. The P-value was calculated by ANOVA with Tukey-Kramer post hoc test.

(F) Intratumoral lactate levels. The P-value was calculated using Kruskal-Wallis test with Steel-Dwass post hoc test.

(G) Quantitative flow cytometric analysis of Foxp-3+ Treg cells. The P-value was calculated using Kruskal-Wallis test with Steel-Dwass post hoc test.

(H) Representative immunohistochemical images of CD8+ T cells and Foxp-3+ T cells in tumors derived from Hepa1-6 cells overexpressing *Ldha* and *Mct4*. Nuclei were stained with hematoxylin. H&E: hematoxylin and eosin. The scale bar represents 200 μm.

(I) Quantitative immunohistochemical analysis of CD8+ T cell and Foxp-3+ T cell infiltration. The P-value was calculated using Kruskal-Wallis test with Steel-Dwass post hoc test.

(J) CD8+ T cell / Foxp-3+ Treg cell ratio. The P-value was calculated using Kruskal-Wallis test with Steel-Dwass post hoc test.

Boxes represent the 25th, 50th, and 75th percentiles. Data are the mean ± SD. n.s, not significant; *p < 0.05, **p < 0.01, ***p < 0.001.

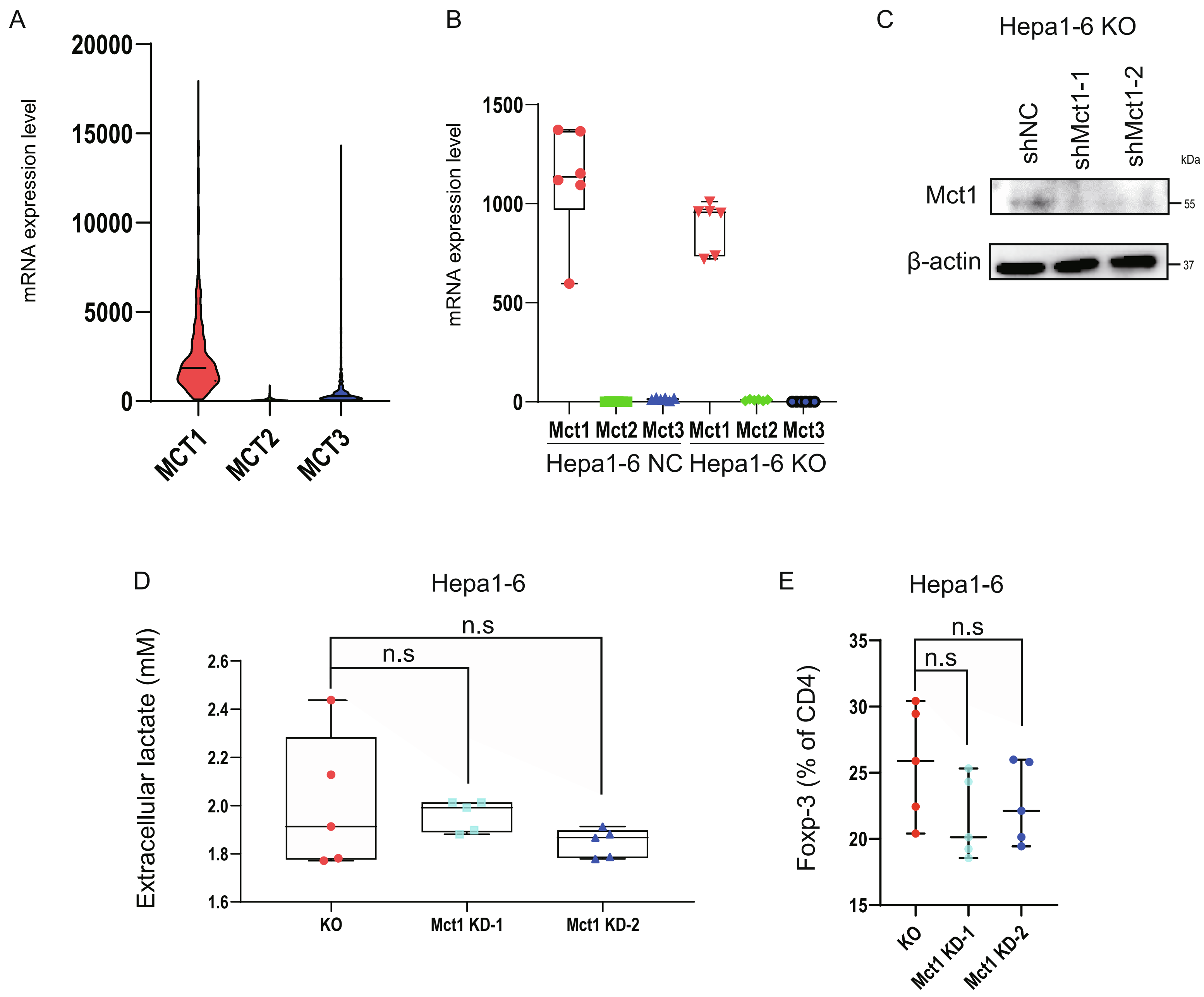

A
mRNA expression level
20000
15000
10000
5000
0
MCT1
MCT2
MCT3
B
mRNA expression level
1500
1000
500
0
Mct1 Mct2 Mct3 Mct1 Mct2 Mct3
Hepa1-6 NC
Hepa1-6 KO
C
Hepa1-6 KO
shNC
shMct1-1
shMct1-2
kDa
Mct1
55
β-actin
37
D
Hepa1-6
n.s
n.s
Extracellular lactate (mM)
2.6
2.4
2.2
2.0
1.8
1.6
KO
Mct1 KD-1
Mct1 KD-2
E
Hepa1-6
n.s
n.s
Foxp-3 (% of CD4)
35
30
25
20
15
KO
Mct1 KD-1
Mct1 KD-2

Supplementary Figure 14. -----related to Figure 6.

(A) Expression analysis of lactate transporters in HCC samples using the TCGA Pancancer Atlas dataset.

(B) Expression analysis of lactate transporters in Hepa1-6 cells.

(C) Western blot analysis of Mct1 in Hepa1-6 KO cells with *Mct1* knockdown. β-Actin was used as an internal control.

(D) Extracellular lactate levels. The P-value was calculated by Kruskal-Wallis test with Steel-Dwass post hoc test.

(E) Quantitative flow cytometric analysis of Foxp-3+ Treg cells co-cultured with Hepa1-6 KO cells with *Mct1* knockdown. The P-value was calculated using Kruskal-Wallis test with Steel-Dwass post hoc test. Boxes represent the 25th, 50th, and 75th percentiles. Data are the mean ± SD. n.s, not significant.

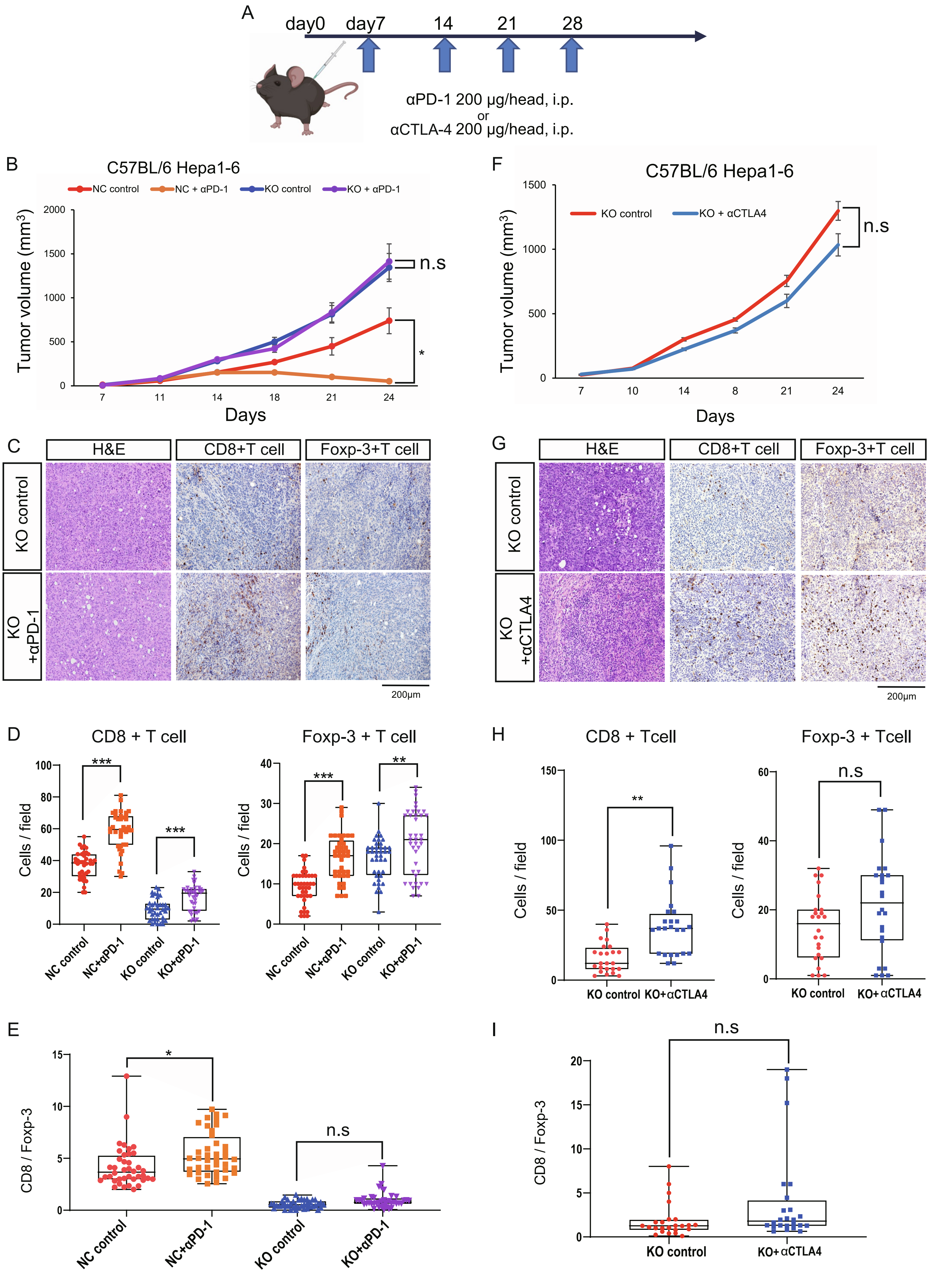
A
day0
day7
14
21
28
αPD-1 200 μg/head, i.p.
or
αCTLA-4 200 μg/head, i.p.
B
C57BL/6 Hepa1-6
NC control
NC + αPD-1
KO control
KO + αPD-1
Tumor volume (mm³)
Days
n.s
*
F
C57BL/6 Hepa1-6
KO control
KO + αCTLA4
n.s
Tumor volume (mm³)
Days
C
H&E
CD8+T cell
Foxp-3+T cell
KO control
KO +αPD-1
200μm
G
H&E
CD8+T cell
Foxp-3+T cell
KO control
KO +αCTLA4
200μm
D
CD8 + T cell
Foxp-3 + T cell
Cells / field
NC control
NC+αPD-1
KO control
KO+αPD-1
***
**
H
CD8 + Tcell
Foxp-3 + Tcell
Cells / field
KO control
KO+αCTLA4
**
n.s
E
CD8 / Foxp-3
NC control
NC+αPD-1
KO control
KO+αPD-1
*
n.s
I
CD8 / Foxp-3
KO control
KO+αCTLA4
n.s

Supplementary Figure 15. -----related to Figure 7.

(A) Schematic representation of anti-PD-1 antibody and anti-CTLA-4 antibody treatment.

(B) Tumorigenicity assay of Hepa1-6 KO cells in immunoproficient mice treated with anti-PD-1 antibody

(N = 4). The P-value was calculated by Welch's *t* test.

(C) Representative immunohistochemical images of CD8+ T cells and Foxp-3+ T cells in tumors derived from Hepa1-6 KO cells treated with anti-PD-1 antibody. Nuclei were stained with hematoxylin. H&E: hematoxylin and eosin. The scale bar represents 200 μm.

(D) Quantitative immunohistochemical analysis of CD8+ T cell and Foxp-3+ T cell infiltration. The P-value was calculated using Kruskal-Wallis test with Steel-Dwass post hoc test.

(E) CD8+ T cell / Foxp-3+ Treg cell ratio. The P-value was calculated using Kruskal-Wallis test with Steel-Dwass post hoc test.

(F) Tumorigenicity assay of Hepa1-6 KO cells in immunoproficient mice treated with anti-CTLA-4 antibody (N = 4). The P-value was calculated by Welch's *t* test.

(G) Representative immunohistochemical images of CD8+ T cells and Foxp-3+ T cells in tumors derived from Hepa1-6 KO cells treated with anti-CTLA-4 antibody. Nuclei were stained with hematoxylin. H&E: hematoxylin and eosin. The scale bar represents 200 μm.

(H) Quantitative immunohistochemical analysis of CD8+ T cell and Foxp-3+ T cell infiltration. The P-value was calculated using Kruskal-Wallis test with Steel-Dwass post hoc test.

(I) CD8+ T cell / Foxp-3+ Treg cell ratio. The P-value was calculated using Kruskal-Wallis test with Steel-Dwass post hoc test.

Boxes represent the 25th, 50th, and 75th percentiles. Data are the mean ± SD. n.s, not significant; *p < 0.05, **p < 0.01, ***p < 0.001. αPD-1: anti-PD-1 antibody. αCTLA-4: anti-CTLA-4 antibody.

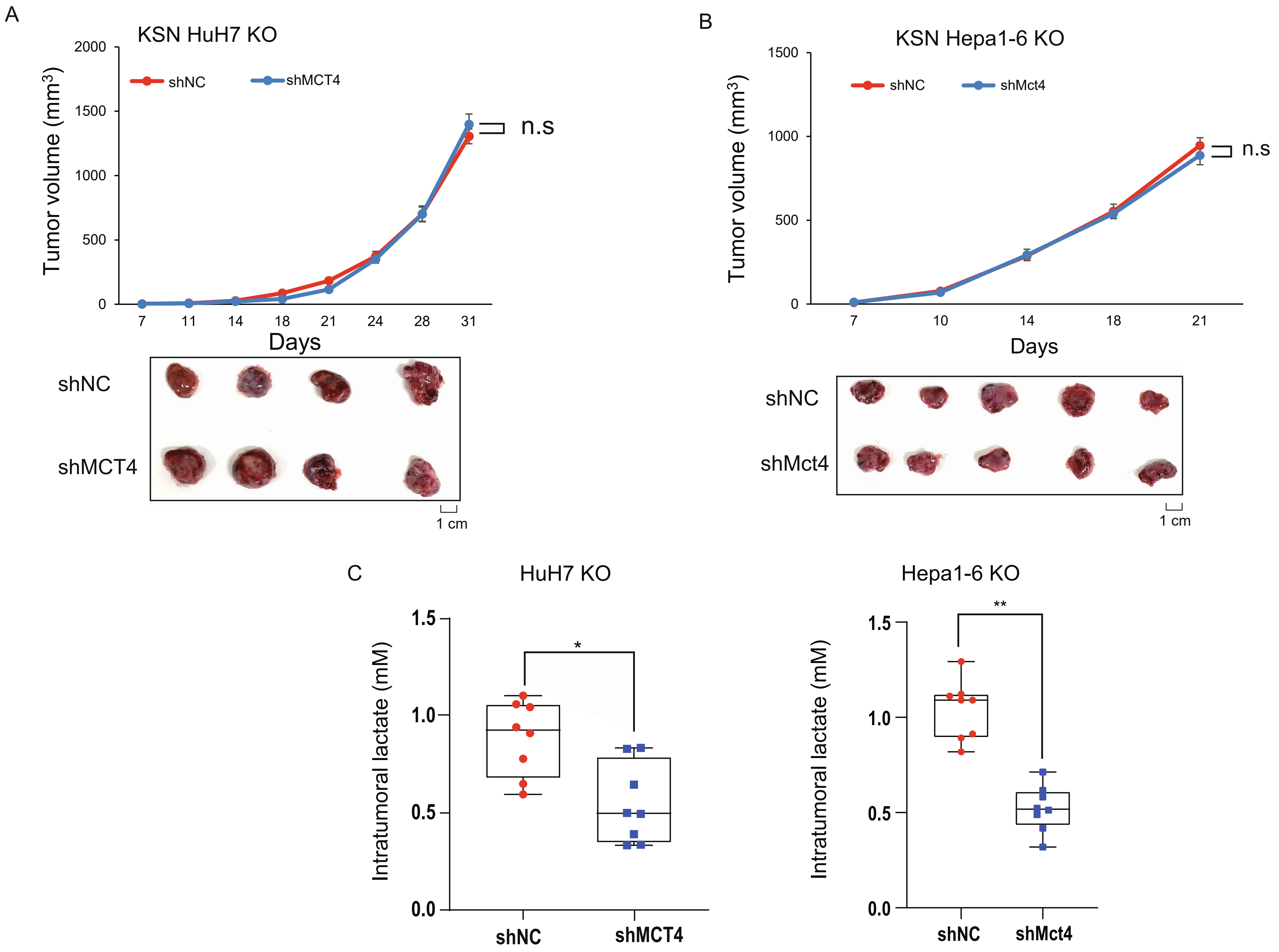

A
KSN HuH7 KO
shNC
shMCT4
Tumor volume (mm3)
2000
1500
1000
500
0
7
11
14
18
21
24
28
31
Days
n.s
shNC
shMCT4
1 cm
B
KSN Hepa1-6 KO
shNC
shMct4
Tumor volume (mm3)
1500
1000
500
0
7
10
14
18
21
Days
n.s
shNC
shMct4
1 cm
C
HuH7 KO
Intratumoral lactate (mM)
1.5
1.0
0.5
0.0
*
shNC
shMCT4
Hepa1-6 KO
Intratumoral lactate (mM)
1.5
1.0
0.5
0.0
**
shNC
shMct4

Supplementary Figure 16. -----related to Figure 7.

(A, B) Tumorigenicity assay of HuH7 KO (A) and Hepa1-6 KO (B) cells with *MCT4* knockdown in immunodeficient mice (N = 4 and 5, respectively), respectively. Representative photo images of tumor specimens were included. The P-value was calculated using Welch's *t* test.

(C) Intratumoral lactate levels in tumors derived from HuH7 and Hepa1-6 cells with *MCT4* knockdown. The P-value was calculated using Mann-Whitney *U* test.

Boxes represent the 25th, 50th, and 75th percentiles. Data are the mean ± SD. n.s, not significant; *p < 0.05, **p < 0.01, ***p < 0.001.

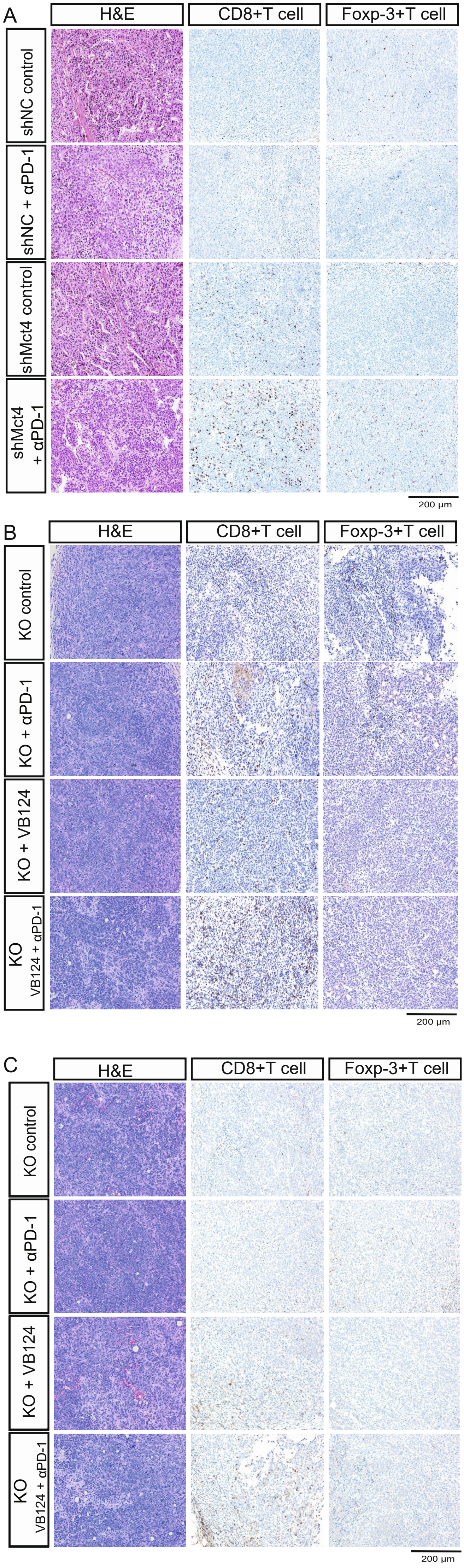
A
H&E
CD8+T cell
Foxp-3+T cell
shNC control
shNC + αPD-1
shMct4 control
shMct4 + αPD-1
200 μm
B
H&E
CD8+T cell
Foxp-3+T cell
KO control
KO + αPD-1
KO + VB124
KO VB124 + αPD-1
200 μm
C
H&E
CD8+T cell
Foxp-3+T cell
KO control
KO + αPD-1
KO + VB124
KO VB124 + αPD-1
200 μm

Supplementary Figure 17. -----related to Figure 7.

(A-C) Representative immunohistochemical images of CD8+ T cells and Foxp-3+ T cells in tumors derived from Hepa1-6 KO cells with *Mct4* knockdown (A), Hepa1-6 KO cells treated with anti-PD-1 antibody and VB124 (B) and 3H3-Pten-KO cells with *Acvr2a* knockout treated with anti-PD-1 antibody. The scale bar represents 200 μm.

(C). Nuclei were stained with hematoxylin. H&E: hematoxylin and eosin.

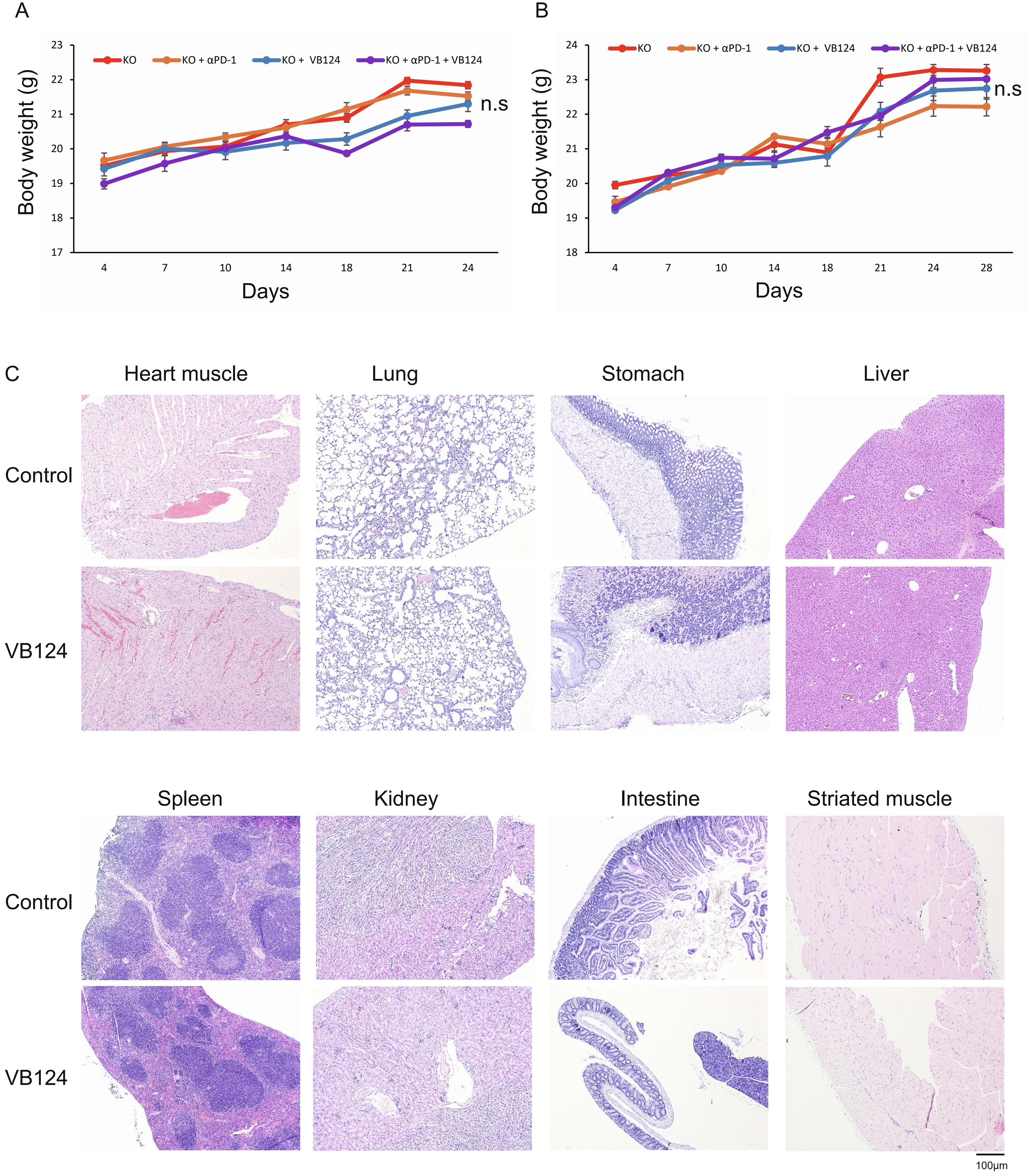
A
B
Body weight (g)
Days
KO
KO + αPD-1
KO + VB124
KO + αPD-1 + VB124
n.s
4
7
10
14
18
21
24
28
17
18
19
20
21
22
23
24
C
Heart muscle
Lung
Stomach
Liver
Control
VB124
Spleen
Kidney
Intestine
Striated muscle
100μm

Supplementary Figure 18. -----related to Figure 7.

(A, B) Body weight of mice treated with anti-PD-1 antibody and VB124. The P-value was calculated using Kruskal-Wallis test with Steel-Dwass post hoc test.

(C) Representative hematoxylin and eosin-stained images of each organ in mice treated with anti-PD-1 antibody and VB124. The scale bar represents 100 μm.

Table S1 is related to Figure 1. Overall survival between vial and non-viral hepatocellular carcinoma.

Gene name	Log-rank analysis	
	Viral	Non-viral
ACVR2A	0.120	0.020*
SPTA1	0.003*	0.004*
BAP1	0.087	0.150
KCNAB1	0.012*	0.002*
PSD2	0.091	0.018*
ARHGAP22	0.024*	0.160
FER1L5	0.170	0.100
HNF1A	0.043*	0.380
NLGN4X	0.037*	0.065
NOS3	0.340	0.120
OR8H2	<0.001*	<0.001*
ZNF727	0.260	0.059
USH2A	0.004*	0.003*
CARD11	0.140	0.008*
TRIM13	0.006*	0.001*
DSCAM	0.031*	0.023*
MLL	0.150	0.097
MYO18B	0.270	0.005*

*indicates significant difference.

Table S2 is related to Figure 2. Top 100 genes with differences are shown.

	Gene.Symnol	log_2FoldChange	Log_{10}P
1	*Krt7*	3.648837	192.3177
2	*Egln3*	3.872062	162.6643
3	*Olfml3*	3.966573	158.9017
4	*Spp1*	2.643132	135.3337
5	*Tff1*	3.977146	111.4696
6	*Avil*	5.138224	109.8528
7	*Gdpd3*	4.505291	109.5444
8	*Aldoa*	2.19755	109.2914
9	*Slc2a3*	3.268018	104.8743
10	*Sema4g*	2.914047	94.74393
11	*Igfbp1*	2.055509	83.18087
12	*Hcfc1r1*	2.478963	80.74165
13	*Bsg*	1.940122	80.56948
14	*Tff2*	1.913875	78.84291
15	*Vegfa*	2.793197	77.06061
16	*Gpi1*	2.039109	75.55229
17	*Ypel3*	3.43984	74.79494
18	*Hsph1*	-2.25391	74.23533
19	*Ero1l*	2.485204	73.62647
20	*Capg*	2.548292	72.08516
21	*Gm35019*	2.824916	71.65967
22	*Ppbp*	2.795174	71.00391
23	*mt-Rnr2*	1.955826	70.59481
24	*Ldha*	1.716446	69.27619
25	*Kctd11*	2.945923	69.16596
26	*Efna1*	3.176839	69.12854
27	*B3gnt3*	2.319278	69.06282
28	*Alb*	1.730494	68.95051
29	*Pgk1*	1.949248	67.59222
30	*H2-Q1*	2.99022	67.3707
31	*Id2*	1.899192	62.98755
32	*Hist1h1c*	3.046714	62.54503
33	*Aplp1*	3.119163	60.6488
34	*Aldoc*	4.206629	60.56418

35	*P4ha1*	2.137269	60.33081
36	*Cdh16*	-3.55184	58.85089
37	*AI506816*	-1.78393	58.18602
38	*Rbp1*	1.963861	58.14242
39	*Lrp1*	1.735902	57.56682
40	*Egln1*	1.933163	56.80535
41	*P4ha2*	2.308552	56.66603
42	*Gys1*	2.075387	56.52741
43	*Slc2a1*	1.85616	54.98706
44	*Ptges*	2.599553	54.14854
45	*Shmt2*	-2.01831	53.93366
46	*Zfpm1*	2.694534	53.60195
47	*Cse1l*	-1.8659	52.96598
48	*Cyp2s1*	1.682417	52.27157
49	*Pfkp*	2.50581	51.83972
50	*mt-Rnr1*	1.780099	51.22786
51	*Hspd1*	-1.62368	50.16398
52	*Pop1*	-2.12878	49.41771
53	*Smox*	1.994287	49.21687
54	*Dhrs3*	2.082031	47.94997
55	*Slc16a3*	1.885928	47.10907
56	*Nrn1*	1.618467	47.00739
57	*Tmem63a*	2.097248	46.44835
58	*Ephx1*	1.506569	46.21927
59	*Cldn4*	-2.48589	46.19829
60	*Csn3*	1.618727	45.93764
61	*Plat*	1.843147	45.50727
62	*Apoc2*	2.373996	45.24658
63	*Apoe*	1.601416	43.99319
64	*Tcof1*	-1.71518	43.86266
65	*Ly6c1*	2.702646	43.63025
66	*Hilpda*	2.06638	42.8041
67	*Mybbp1a*	-1.4989	41.79239
68	*Kif21b*	4.003562	41.1948
69	*Acap1*	4.647406	41.02339
70	*Heatr1*	-2.40121	40.77671

71	*Selenbp1*	4.437324	40.48204
72	*Azin1*	-1.90361	40.15173
73	*Tpi1*	1.364265	40.11468
74	*Pdgfa*	1.907091	39.80499
75	*Cluh*	-1.56992	39.74174
76	*Dusp1*	2.116022	39.02045
77	*Unc13d*	5.349676	38.81163
78	*Pgm1*	1.858117	38.68839
79	*Cdc42ep2*	2.982175	38.68057
80	*Sdc4*	1.878168	38.49873
81	*Hcfc1*	-1.50978	37.98037
82	*Nol6*	-1.64906	37.84702
83	*Cd68*	2.226466	37.82263
84	*Gm16439*	5.177313	37.76642
85	*Gm26883*	2.547419	37.52004
86	*Id1*	1.575484	37.40941
87	*Gpam*	-3.11415	37.28867
88	*Mapk8ip3*	1.59324	36.94149
89	*Kpna2*	-1.49512	36.43275
90	*Mcm2*	-1.43478	36.3929
91	*Rcor2*	2.373483	36.12316
92	*Gulo*	3.534626	36.04865
93	*Mxi1*	2.133162	35.91742
94	*Clu*	1.302494	35.82656
95	*Camk2n1*	1.987645	34.9386
96	*Hk1*	1.384809	34.86229
97	*Ramp3*	2.5066	34.66999
98	*Ddx21*	-1.93679	34.60416
99	*Fn1*	1.413631	34.44608
100	*Rrp12*	-2.46736	34.38463

Table S3. Patients' characteristics, preoperative general conditions, and tumor factors.

	ACVR2A-high	ACVR2A-low	
Variables	(N = 156)	(N = 38)	P-value
Age*	71 (44–88)	73 (37–93)	0.149
Sex (male:female)	123:33	29:9	0.736
Preoperative co-morbidity			
Hypertension	62 (40)	15 (39)	0.953
Diabetes mellitus	59 (38)	15 (39)	0.873
Hyperlipidemia	28 (18)	5 (24)	0.439
Alcohol consumption			0.045
Nothing	71 (40)	8 (21)	
Moderate	33 (21)	14 (37)	
Excess	28 (18)	10 (26)	
Background of hepatitis			0.027
B hepatitis	25 (16)	5 (13)	**0.011
C hepatitis	60 (38)	7 (18)	
NBNC	71 (46)	26 (68)	
MASH	34 (22)	15 (39)	0.024
Foxp-3 positive	28 (18)	24 (63)	<0.001
LDHA strong	36 (23)	26 (68)	<0.001
BMI*	23 (15-36)	23 (17-32)	0.357
Serum Alb (g/dL)*	4.0 (3.0-4.9)	4.0 (2.3-4.7)	0.689
Serum AST (IU/L)*	33 (13-231)	30 (13-84)	0.496
Serum ALT (IU/L)*	27 (7-186)	23 (8-122)	0.201
Platelet count (10^4/mL)*	15.8 (4.9-39.6)	14.9 (7.2-52.1)	0.951
Fib 4 index*	2.61 (1.33-18.3)	3.30 (0.69-8.10)	0.388
ALBI grade			0.141
1	87 (56)	25 (66)	
2a	47 (30)	7 (18)	
2b	22 (14)	5 (13)	
3	0 (0)	1 (5)	
ICGR15 (%)*	12.7 (2.9-87.5)	11.5 (4.3-30.7)	0.264
AFP > 100 (mg/dl)*	45 (29)	13 (34)	0.661
DCP > 400 (mAU/ml)*	51 (33)	13 (34)	0.891
Child-Pugh classification			0.006

A	153 (98)	33 (86)	
B or C	3 (2)	5 (13)	
Number of tumor			0.022
Single	120 (77)	22 (58)	
Multiple	36 (23)	16 (42)	
Tumor size (cm)*	3.5 (0.8-20.0)	4.0 (1.0-19.0)	0.051
Differentiation			0.669
High	56 (36)	11 (29)	
Middle	81 (52)	21 (55)	
Low	19 (12)	6 (16)	
Portal vein invasion			0.108
0	110 (71)	20 (52)	
1	38 (24)	15 (39)	
2	6 (4)	1 (2)	
3	1 (1)	0 (0)	
4	1 (1)	2 (5)	
Hepatic vein invasion			0.607
0	142 (91)	32 (84)	
1	11 (7)	4 (11)	
2	2 (1)	1 (2)	
3	1 (1)	1 (2)	
Hepatic artery invasion			0.349
0	154 (99)	38 (100)	
1	2 (1)	0 (0)	
Bile duct invasion			0.087
0	152 (97)	35 (92)	
1	2 (1)	3 (8)	
2	2 (1)	0 (0)	
Fibrosis stage			0.985
f0	25 (16)	7 (18)	
f1	29 (19)	7 (18)	
f2	30 (19)	7 (18)	
f3	30 (19)	6 (16)	
f4	42 (27)	11 (29)	
Non-cancerous part of tissue			0.864

NL	23 (15)	7 (18)	
LF/CH	86 (56)	20 (53)	
LC	46 (30)	11 (29)	

Values in parentheses are percentages unless indicated otherwise; *values are median (range). **compared between viral hepatitis and NBNC. ***compared between ALBI grade 1 and grade 2-3. ACVR2A, activin receptor type II A; NBNC, non-HBV, non-HCV; MASH, metabolic dysfunction associated steatohepatitis; Foxp-3, forkhead box protein 3, human nomenclature; LDHA, lactate dehydrogenase A; BMI, body mass index; ALBI, albumin-bilirubin; ICGR-15, indocyanine green retention rate at 15 min; AFP, α-fetoprotein; DCP, des-γ-carboxy prothrombin. P-value was calculated by Welch's t-test (parametric) or Fisher's exact test (non-parametric). NL, normal liver; LF/CH, liver fibrosis/chronic hepatitis; LC, liver cirrhosis.

P-value was calculated by Welch's t-test (parametric) or Fisher's exact test (non-parametric)

Table S4 is related to Figure 7. Patients' characteristics and preoperative general conditions

Variables	FDG-PET hyperaccumulation (N = 29)	FDG-PET hypoaccumulation (N = 15)	P-value
Age*	71 (57–80)	74 (56–85)	0.393
Sex (male:female)	23:6	15:1	0.174
ACVR2A low	8 (28)	1 (6)	0.066
Foxp-3 positive	9 (31)	1 (6)	0.039
LDHA strong	15 (52)	2 (13)	0.006

Values in parentheses are percentages unless indicated otherwise; *values are median (range).

FDG-PET, 18F-fluorodeoxyglucosepositron positron emission tomography; ACVR2A, activin receptor type IIA; Foxp-3, forkhead box protein 3; LDHA, lactate dehydrogenase A.

P-value was calculated by Welch's t-test (parametric) or Fisher's exact test (non-parametric)

Table S5 is related to STAR Methods. Primers used in the study.

Gene ID	name	sequence
92	hACVR2A-F	GTTTGCCGTCTTTCTTATCTCCT
	hACVR2A-R	GTCACCATAACACGGTTCAACA
11480	mAcvr2a-F	GCGTTCGCCGTCTTTCTTATC
	mAcvr2a-R	GTTGGTTCTGTCTCTTTCCCAAT
3939	hLDHA-F	ATGGCAACTCTAAAGGATCAGC
	hLDHA-R	CCAACCCCAACAACTGTAATCT
16828	mLdha-F	TGTCTCCAGCAAAGACTACTGT
	mLdha-R	GACTGTACTTGACAATGTTGGGA
2597	hGAPDH-F	GGAGCGAGATCCCTCCAAAAT
	hGAPDH-R	GGCTGTTGTCATACTTCTCATGG
14433	mGapdh-F	AGGTCGGTGTGAACGGATTTG
	mGapdh-R	TGTAGACCATGTAGTTGAGGTCA
226	hALDOA-F	CGTGTGAATCCCTGCATTGG
	hALDOA-R	CAGCCCCTGGGTAGTTGTC
11674	mAldoa-F	CGTGTGAATCCCTGCATTGG
	mAldoa-R	CAGCCCCTGGGTAGTTGTC
230	hALDOC-F	AGAAGGAGTTGTCGGATATTGCT
	hALDOC-R	TTCTCCACCCCAATTTGGCTC
11676	mAldoc-F	AGAAGGAGTTGTCGGATATTGCT
	mAldoc-R	TTCTCCACCCCAATTTGGCTC
2023	hENO1-F	TGGTGTCTATCGAAGATCCCTT
	hENO1-R	CCTTGGCGATCCTCTTTGG
13806	mEno1-F	TGCGTCCACTGGCATCTAC
	mEno1-R	CAGAGCAGGCGCAATAGTTTTA
5230	hPGK1-F	TGGACGTTAAAGGGAAGCGG
	hPGK1-R	GCTCATAAGGACTACCGACTTGG
18655	mPgk1-F	ATGTCGCTTTCCAACAAGCTG
	mPgk1-R	GCTCCATTGTCCAAGCAGAAT
6513	hSLC2A1-F	GGCCAAGAGTGTGCTAAAGAA
	hSLC2A1-R	ACAGCGTTGATGCCAGACAG
20525	mSlc2a1-F	CAGTTCGGCTATAACACTGGTG
	mSlc2a1-R	GCCCCCGACAGAGAAGATG
5310	hPKD1-F	AGACCAACGATACCCTGTTCT
	hPKD1-R	GGCTGTACCTCACTAGGACTC
18763	mPkd1-F	CTAGACCTGTCCCACAACCTA
	mPkd1-R	GCAAACACGCCTTCTTCTAATGT
7422	hVEGFA-F	AGGGCAGAATCATCACGAAGT
	hVEGFA-R	AGGGTCTCGATTGGATGGCA
22339	mVegfa-F	GCACATAGAGAGAATGAGCTTCC

	mVegfa-R	CTCCGCTCTGAACAAGGCT
3091	hHIF1A-F	GAACGTCGAAAAGAAAAGTCTCG
	hHIF1A-R	CCTTATCAAGATGCGAACTCACA
15251	mHif1a-F	ACCTTCATCGGAAACTCCAAAG
	mHif1a-R	CTGTTAGGCTGGGAAAAGTTAGG
9123	hSLC16A3-F	CCATGCTCTACGGGACAGG
	hSLC16A3-R	GCTTGCTGAAGTAGCGGTT
80879	mSlc16a3-F	TCACGGGTTTCTCCTACGC
	mSlc16a3-R	GCCAAAGCGGTTCACACAC
19791	18S-F	TAGAGTGTTCAAAGCAGGCCC
	18S-R	CCAACAAAATAGAACCGCGGT
